# Supplementary material for: Neighbourhood socioeconomic characteristics and blood pressure among Jamaican youth: a pooled analysis of data from observational studies
Source: PeerJ. 2020 Oct 6;8:e10058. doi: 10.7717/peerj.10058 (PMC7546221; doi:10.7717/peerj.10058)
Supplement: Supplemental Information 1 [file peerj-08-10058-s001.pdf]

NEIGHBOURHOOD SOCIOECONOMIC CHARACTERISTICS AND BLOOD PRESSURE AMONG JAMAICAN YOUTH: A POOLED ANALYSIS OF DATA FROM OBSERVATIONAL STUDIES

AUTHORS:

Trevor S Ferguson[1], Novie OM Younger-Coleman[1], Jasneth Mullings[2], Damian Francis[3], Lisa-Gaye Greene[4], Parris Lyew-Ayee[4], Rainford Wilks[1]

INSTITUTIONS:

1. Epidemiology Research Unit, Caribbean Institute for Health Research, The University of the West Indies, Mona, Kingston 7, Jamaica
2. Health Research Resource Unit, Dean's Office, Faculty of Medical Sciences, University of the West Indies, Mona, Kingston 7, Jamaica
3. School of Health and Human Performance, Georgia College and State University, Milledgeville, GA, USA
4. Mona GeoInformatics Institute, The University of the West Indies, Mona, Kingston 7, Jamaica

SUPPLEMENTARY TABLES

Table S1: Primary data sources for community characteristics

Characteristics	Data Sources
Included in PCA Score	
Poverty (%)	Planning Institute of Jamaica 2001/2002
Unemployment (%)	Population Census 2011; Statistical Institute of Jamaica
Dependency ratio	Mona Geo-Informatics Institute
Population density (persons/km^2)	Planning Institute of Jamaica 2001/2002; Statistical Institute of Jamaica; Population Census 2011;
House Size (% ≥2 bedrooms/number of households)	Mona Geo-Informatics Institute
Tertiary Education Level (%)	Mona Geo-Informatics Institute
**Other Community Characteristics**	
Population (number of persons)	Population Census 2011; Statistical Institute of Jamaica
Secondary Education Level (%)	Mona Geo-Informatics Institute
Primary Education Level (%)	Mona Geo-Informatics Institute
Murder Rates (number/1000 pop)	Jamaica Constabulary Force; Mona Geo-Informatics Institute

All data were obtained from the Mona Geo-Informatics Institute; the Institute harnesses data from several primary sources as indicated above.

Table S2: Summary statistics for participant and community characteristics by study

Characteristics	1986 Birth Cohort n = 893 Median (p25, p75)	Youth Risk Survey n = 1200 Median (p25, p75)	Jamaica Health & Lifestyle Survey n = 463 Median (p25, p75)	P-value for difference in median across studies
Included in PCA Score				
Poverty (%)	12.9 (6.7, 22.0)	17.8 (9.8, 26.9)	16.9 (10.8, 24.6)	<0.001
Unemployment (%)	8.1 (6.8, 10.2)	7.7 (6.5, 9.9)	8.3 (6.3, 10.2)	0.201
Dependency ratio	0.68 (0.60, 0.75)	0.75 (0.64, 0.84)	0.75 (0.65, 0.81)	<0.001
Population density (persons/km^2)	1776 (1237, 5499)	502 (190, 3880)	541 (211, 1419)	<0.001
House Size (% ≥2 bedrooms/number of households)	7.4 (6.1, 10.6)	7.5 (6.3, 10.0)	7.5 (6.4, 9.3)	0.226
Tertiary Education Level (%)	10.1 (5.9, 21.7)	5.9 (3.7, 13.1)	6.1 (3.4, 12.6)	<0.001
**Other Community Characteristics**				
Population (number of persons)	7780 (4572, 12183)	5544 (2782, 8837)	4436 (2463, 10157)	<0.001
Secondary Education Level (%)	49.0 (46.2, 54.2)	48.0 (45.4, 51.3)	47.6 (44.0, 51.1)	<0.001
Primary Education Level (%)	25.6 (21.0, 29.2)	32.6 (25.3, 38.8)	32.2 (26.4, 38.0)	<0.001
Murder Rates (number/1000 pop)	1.98 (1.10, 3.65)	1.15 (0.49, 2.77)	1.40 (0.47, 2.13)	<0.001

p25 = 25th centile, p75 = 75th centile
The 1986 birth cohort included 169 communities, Youth Risk survey 132 communities and Jamaica Health and Lifestyle Survey 117 communities
P-values were obtained from the non-parametric test for equality of medians test in Stata; the test reports p-values from Chi-squared tests comparing the distribution of values above and below the median in each study group.

Table S3: Eigenvectors (factor loadings) for the individual variables in PCA derived SES components 1 and 2

**Variable**	**PCA Component 1**	**PCA Component 2**
Poverty	-0.4839	-0.0504
Unemployment (%)	-0.1410	0.7078
Dependency ratio	-0.4991	0.0380
Population density (persons per km^2)	0.3524	0.4520
House Size (% ≥2 bedroom per household)	0.3563	-0.5135
Tertiary Education (%)	0.4957	-0.1645

Overall KMO = 0.7744
PCA = Principal Component Analysis; SES = socioeconomic status; KMO = Kaiser-Meyer-Olkin test for sampling adequacy

Table S4: Number of Missing Values for Variables Included in the Analysis

Variable	Number Missing	Number Non-Missing	% Missing
Age	1	2,555	0.04
Weight	4	2,552	0.16
Height	8	2,548	0.31
Body mass index	10	2,546	0.39
Waist circumference	10	2,546	0.39
Fasting Glucose	144	2,412	5.63
Fasting Cholesterol	152	2,404	5.95
Household Possessions	1	2,555	0.04
Physical Activity Level	1	2,555	0.04
Fast food consumption	23	2,533	0.90
All Variables	231	2325	9.04

Table S5: Coefficients for assessment of sex interaction in relationship between systolic blood pressure and participant characteristics

Characteristics	Males N=1110 B-coefficient (95% CI)	Females n=1446 B-coefficient (95% CI)	P-value for interaction by sex
PCA SES Component 1 (z-score)	-1.45 (-1.82, -1.07)	-1.30 (-2.16, -0.44)	0.776
PCA SES Component 1 (z-score)			
Spline 1 (z-score < -1)	1.73 (-0.14, 3.59)	2.74 (0.29, 5.20)	0.572
Spline 2 (z-score -1 to 0)	2.58 (0.41, 4.76)	3.51 (2.28, 4.75)	0.505
Spline 3 (z-score >0 to 1)	0.55 (-1.98, 3.08)	0.02 (-1.82, 1.86)	0.873
Spline 4 (z-score >1)	0.11 (-0.15, 0.38)	-0.46 (-0.87, -0.05)	0.001
Age (years)	1.08 (0.64, 1.54)	0.40 (0.002, 0.79)	<0.001
Body mass index (z-score)	2.63 (2.01, 3.26)	1.87 (1.47, 2.27)	0.038
Fasting glucose (mmol/l)	1.24 (-0.15, 2.63)	0.45 (0.02, 0.88)	0.156
Household Possessions Category			
Lower Third	Reference category	Reference category	
Middle Third	-1.11 (-2.22, -0.002)	-0.46 (-1.31, 0.39)	0.502
Upper Third	-0.84 (-2.88, 1.20)	-1.59 (-2.60, -0.58)	0.381
Fast Food consumption			
<= 2 times/week	Reference category	Reference category	
3-4 times/week	0.46 (-0.79, 1.72)	-1.38 (-2.34, -0.42)	0.039
>= 5 times/week	-0.86 (-2.93, 1.21)	-0.43 (-1.63, 0.78)	0.920
Physical Activity			
High	Reference category	Reference category	
Moderate	0.24 (-1.01, 1.49)	-0.06 (-1.71, 1.59)	0.694
Low	-1.57 (-3.34, 0.20)	-0.24 (-1.60, 1.13)	0.413

PCA = Principal Components Analysis. SES = socioeconomic status

P-values are for tests for interaction in three level linear mixed effect models in Stata. Level 1 – individual study participants; level 2 – communities in which participant lived; level 3 – parish in which community was situated. Models were run on the first imputed dataset and used robust standard errors.

Table S6: Summary statistics for participant and community characteristics by study and sex

Characteristics	1986 Birth Cohort		Youth Risk Survey		Jamaica Health & Lifestyle Survey	
	Female<br>n = 485	Male<br>n = 408	Female<br>n = 657	Male<br>n = 543	Female<br>n = 304	Male<br>n = 159
Participant Characteristics	**Mean (SD)**	**Mean (SD)**	**Mean (SD)**	**Mean (SD)**	**Mean (SD)**	**Mean (SD)**
Age (years)	18.8 (0.6)	18.8 (0.6)	16.5 (1.3)	16.6 (1.3)	20.0 (2.8)	19.6 (2.6)
Height (cm)	163.6 ± 4.4	176.8 ± 5.4***	162.8 ± 6.2	172.1 ± 5.5***	163.5 ± 4.6	174.6 ± 7.5***
Weight (kg)	62.4 (15.5)	71.1 (14.2) ***	59.6 (12.7)	65.0 (12.7) ***	65.5 (15.7)	70.1 15.5) **
Body mass index (kg/m^2)	23.3 (5.6)	22.7 (4.3)	22.6 (5.0)	22.0 (4.3) *	24.5 (5.7)	23.1 (5.9) *
Systolic blood pressure (mmHg)	107.4 (8.8)	113 (10.4) ***	109.7 (9.6)	113.4 (11.3) ***	111.3 (11.3)	118.8 12.6) ***
Diastolic blood pressure (mmHg)	66.9 (9.3)	69.2 (10.4) ***	70.0 (9.6)	70.9 (10.7) *	70.7 (10.2)	73.9 (10.7) **
Waist circumference (cm)	73.9 (12.1)	75.2 (10.9)	71.2 (9.9)	72.2 (7.9) *	77.8 (14.5)	75.8 (9.4)
Fasting glucose (mmol/l)	4.4 (0.4)	4.7 (0.6) ***	3.5 (1.3)	3.5 (1.1)	3.9 (1.1)	4.3 (2.3) *
PCA-SES Component 1 (z-score)	0.38 (1.0)	0.35 (0.96)	-0.22 (0.97)	-0.11 (1.0)	-0.21 (0.84)	-0.16 (0.89)
PCA SES Component 2 (z-score)	0.05 (1.16)	0.13 (1.14)	-0.03 (0.85)	0.01 (1.01)	-0.13 (0.87)	-0.08 (0.96)
	**n (%)**	**n (%)**	**n (%)**	**n (%)**	**n (%)**	**n (%)**
Elevated BP/HTN (BP≥120/80 mmHg)	66 (13.6)	122 (29.9) ***	175 (26.6)	213 (39.2) ***	93 (30.6)	80 (50.3) ***
Body Mass Index Category		***		***		***
Underweight	69 (14.3)	25 (6.1)	102 (15.6)	79 (14.6)	35 (11.7)	12 (7.6)
Normal weight	268 (55.5)	307 (75.3)	394 (60.1)	382 (70.5)	150 (50.2)	114 (72.2)
Overweight	96 (19.9)	53 (13.0)	113 (17.2)	56 (4.6)	62 (20.7)	20 (12.7)
Obesity	50 (10.4)	23 (5.6)	47 (7.2)	25 (4.6)	52 (17.4)	12 (7.6)
Household Possessions Category						
Lower Third	201 (41.4)	149 (36.1)	244 (37.1)	173 (31.9)	97 (31.9)	49 (30.8)
Middle Third	161 (33.2)	139 (34.1)	236 (35.9)	201 (37.0)	107 (35.2)	50 (31.2)
Upper Third	123 (25.4)	119 (29.2)	177 (26.9)	169 (31.1)	100 (32.9)	60 (37.4)

Continued on next page

Table S6: Summary statistics for participant and community characteristics by study and sex, continued

Characteristics	1986 Birth Cohort		Youth Risk Survey		Jamaica Health & Lifestyle Survey	
	Female n = 485	Male n = 408	Female n = 657	Male n = 543	Female n = 304	Male n = 159
Fast Food consumption						
<= 2 times/week	379 (78.5)	308 (77.4)	537 (82.5)	467 (86.8)	228 (75.0)	126 (79.3)
3-4 times/week	68 (14.1)	54 (13.6)	65 (10.0)	40 (7.4)	52 (17.1)	23 (14.5)
>= 5 times/week	36 (7.5)	36 (9.1)	49 (7.5)	31 (5.8)	24 (7.9)	10 (6.3)
Physical Activity		***		***		***
High	62 (12.8)	153 (37.6)	234 (35.6)	330 (60.8)	44 (14.5)	79 (26.6)
Moderate	193 (39.8)	180 (44.2)	174 (26.5)	93 17.1)	67 (22.0)	40 (25.2)
Low	230 (47.4)	74 (18.2)	249 (37.9)	120 (22.1)	193 (63.5)	40 (25.2)

*p<0.05; **p<0.01; ***P<0.001 for sex differences within studies derived from t-tests for continuous variables and Chi-squared tests for categorical variables. SD = standard deviation.

Table S7: Sex specific P-values for interaction by study for neighbourhood SES variables

Neighbour SES Variable	Males		Females	
	Youth Risk Survey	JHLS-II	Youth Risk Survey	JHLS-II
PCA SES Component 1 (z-score)	0.846	0.977	0.832	0.862
PCA SES Component 1 (z-score)				
Spline 1 (z-score < -1)	<0.001	0.328	0.300	0.700
Spline 2 (z-score -1 to 0)	0.308	0.587	0.135	0.751
Spline 3 (z-score >0 to 1)	0.184	0.688	<0.001	0.657
Spline 4 (z-score >1)	<0.001	0.002	0.023	0.166

The 1986 Jamaica Birth Cohort study was the comparator for these analyses.
P-values are for tests for interaction in three level linear mixed effect models in Stata. Level 1 – individual study participants; level 2 – communities in which participant lived; level 3 – parish in which community was situated. Models were run on the first imputed dataset and used robust standard errors

Table S8: Odd ratio for association with elevated blood pressure or hypertension for individual characteristics for male and female participants in bivariate regression model

Characteristics	Males N=1110 Odds Ratio (95% CI)	P-value	Females n=1446 Odds ratio (95% CI)	P-value
PCA SES Component 1 (Thirds)				
Lower Third	Reference category		Reference category	
Middle Third	0.77 (0.56, 1.06)	0.109	0.86 (0.60, 1.23)	0.411
Upper Third	0.65 (0.46, 0.92)	0.015	0.79 (0.52, 1.20)	0.263
PCA SES Component 1 (Thirds)				
Lower Third	Reference category		Reference category	
Middle Third	1.19 (0.85, 1.66)	0.302	1.36 (0.93, 1.98)	0.109
Upper Third	0.95 (0.68, 1.32)	0.747	1.09 (0.72, 1.65)	0.680
Age categories				
15-17 years	Reference category		Reference category	
18-19 years	1.04 (0.77, 1.41)	0.796	0.91 (0.67, 1.24)	0.556
20-24 year	1.51 (0.98, 2.31)	0.061	1.52 (1.03, 2.26)	0.036
Body mass index categories				
Underweight	0.76 (0.49, 1.18)	0.217	0.76 (0.49, 1,17)	0.211
Normal weight	Reference category		Reference category	
Overweight	1.32 (0.89, 1.99)	0.170	1.73 (1.24, 2.43)	0.001
Obesity	4.53 (2.54, 8.09)	<0.001	2.71 (1.81, 4.06)	<0.001
High glucose (upper quintile)	1.64 (1.21, 2.22)	0.001	1.17 (0.80, 1.70)	0.424
Household Possessions Category				
Lower Third	Reference category		Reference category	
Middle Third	0.98 (0.72, 1.32)	0.874	1.14 (0.86, 1.54)	0.391
Upper Third	0.94 (0.68, 1.28)	0.679	0.72 (0.51, 1.02)	0.066
Fast Food consumption				
<= 2 times/week	Reference category		Reference category	
3-4 times/week	0.99 (0.65, 1.51)	0.971	0.74 (0.49, 1.11)	0.145
>= 5 times/week	1.04 (0.63, 1.73)	0.875	0.67 (0.39, 1.16)	0.154
Physical Activity				
High	Reference category		Reference category	
Moderate	0.72 (0.53, 0.97)	0.031	1.22 (0.85, 1.75)	0.273
Low	0.70 (0.50, 0.98)	0.036	0.96 (0.69, 1.34)	0.804

PCA = Principal Components Analysis. SES = socioeconomic status

P-values from three level logistic mixed effect models in Stata. Level 1 – individual study participants; level 2 – communities in which participant lived; level 3 – parish in which community was situated. Models used multiple imputation to account for missing data.

Table S9: Complete case analysis: multivariable models for association between systolic blood pressure and covariates for male and female participants

Characteristics	Males β-coefficient (95% CI) n = 1019		Females β-coefficient (95% CI) n = 1362	
PCA SES Component 1 (z-score)	-1.32 (-1.77, -0.85)	<0.001	-0.42 (-1.56, 0.71)	0.465
PCA SES Component 2 (linear splines)				
Spline 1 (z-score < -1)	0.23 (-3.71, 4.16)	0.909	-0.23 (-2.53, 2.07)	0.846
Spline 2 (z-score -1 to 0)	1.18 (-2.05, 4.42)	0.474	4.10 (1.59, 6.62)	0.001
Spline 3 (z-score >0 to 1)	-0.89 (-3.40, 1.62)	0.488	-3.07 (-5.50, -0.64)	0.013
Spline 4 (z-score >1)	0.65 (-0.82, 1.39)	0.082	0.62 (-1.08, 2.33)	0.475
Age (years)	0.56 (0.07, 1.18)	0.083	0.30 (-0.10, 0.70)	0.137
Household Possessions Category				
Lower Third	Reference category		Reference category	
Middle Third	-1.37 (-2.28, -0.47)	0.003	-0.68 (-1.34, -0.02)	0.045
Upper Third	-1.33 (-3.51, 0.84)	0.229	-1.70 (-2.47, -0.93)	<0.001
Study				
1986 Birth Cohort	Reference category		Reference category	
Youth Risk Survey	-0.34 (-5.69, 5.01)	0.901	2.20 (0.31, 4.10)	0.023
JHLS-II	2.07 (-1.67, 5.80)	0.278	3.16 (2.08, 4.24)	<0.001
Fasting glucose (mmol/l)	0.98 (-0.16, 2.12)	0.093	0.45 (-0.08, 0.97)	0.098
Body mass index (z-score)	2.78 (1.84, 3.70)	<0.001	1.79 (1.38, 2.21)	<0.001
Physical Activity				
High	Reference category		Reference category	
Moderate	-0.30 (-2.06, 1.47)	0.742	0.71 (-1.25, 2.68)	0.475
Low	-1.33 (-2.90, 0.23)	0.095	0.19 (-1.28, 1.66)	0.803
Fast Food consumption				
<= 2 times/week	Reference category		Reference category	
3-4 times/week	0.08 (-1.86, 2.01)	0.938	-0.61 (-1.67, 0.45)	0.258
>= 5 times/week	-0.88 (-3.10, 1.34)	0.438	0.09 (-1.31, 1.49)	0.900

PCA = Principal Components Analysis; SES = socioeconomic status

Model adjusted for age, household SES, study, BMI z-score, glucose, physical activity, and fast food consumption

P-values from three level linear mixed effect models in Stata. Level 1 – individual study participants; level 2 – communities in which participant lived; level 3 – parish in which community was situated. Models used multiple imputation to account for missing data and used robust standard errors.

Table S10: Complete case analysis: multivariable models for association between diastolic blood pressure and covariates for male and female participants

Characteristics	Males<br>β-coefficient (95% CI)<br>n = 1019		Females<br>β-coefficient (95% CI)<br>n = 1362	
PCA SES Component 1 (z-score)	-0.12 (-0.72, 0.48)	0.700	0.73 (-0.45, 1.90)	0.226
PCA SES Component 2 (linear splines)				
Spline 1 (z-score < -1)	-3.84 (-6.25, -1.44)	0.002	1.23 (0.30, 2.17)	0.010
Spline 2 (z-score -1 to 0)	3.41 (1.64, 5.18)	<0.001	0.37 (-1.62, 2.37)	0.715
Spline 3 (z-score >0 to 1)	-2.56 (-5.08, -0.04)	0.047	-0.93 (-3.30, 1.44	0.443
Spline 4 (z-score >1)	0.79 (-0.002, 1.58)	0.051	-0.09 (-1.20, 1.03)	0.875
Age (years)	0.63 (0.16, 1.10)	0.009	0.19 (0.01, 0.37)	0.034
Household Possessions Category				
Lower Third	Reference category		Reference category	
Middle Third	-0.67 (-2.26, 0.92)	0.410	-0.22 (-1.01, 0.56)	0.578
Upper Third	-0.68 (-2.02, 0.66)	0.319	-0.87 (-2.21, 0.47)	0.203
Study				
1986 Birth Cohort	Reference category		Reference category	
Youth Risk Survey	1.91 (-2.27, 6.08)	0.371	3.10 (1.81, 4.39)	<0.001
JHLS-II	2.72 (1.26, 4.18)	<0.001	3.71 (1.61, 5.81)	0.001
Fasting glucose (mmol/l)	0.31 (-0.63, 1.26)	0.516	0.54 (0.08, 0.99)	0.021
Body mass index (z-score)	0.38 (-0.78, 1.56)	0.525	0.78 (0.29, 1.27)	0.002
Physical Activity				
High	Reference category		Reference category	
Moderate	0.36 (-1.66, 2.36)	0.730	0.59 (-0.13, 1.32)	0.110
Low	-1.04 (-2.80, 0.71)	0.245	-0.65 (-1.46, 0.15)	0.113
Fast Food consumption				
<= 2 times/week	Reference category		Reference category	
3-4 times/week	0.67 (-0.89, 2.21)	0.401	0.56 (-0.60, 1.72)	0.347
>= 5 times/week	1.14 (-0.61, 2.90)	0.202	-0.85 (-2.21, 0.51)	0.220

PCA = Principal Components Analysis; SES = socioeconomic status

Model adjusted for age, household SES, study, BMI z-score, glucose, physical activity, and fast food consumption

P-values from three level linear mixed effect models in Stata. Level 1 – individual study participants; level 2 – communities in which participant lived; level 3 – parish in which community was situated. Models used multiple imputation to account for missing data and used robust standard errors.

Table S11: Multivariable models for association between systolic blood pressure and neighbourhood socioeconomic status for male and female participants in each study

Characteristics	1986 Birth Cohort		Youth Risk Survey		JHLS-II	
**MALES**	**β (95% CI)**	**p-value**	**β (95% CI)**	**p-value**	**β (95% CI)**	**p-value**
PCA SES Component 1 (z-score)	-0.54 (-1.15, 0.06)	0.080	-0.66 (-1.89, 0.57)	0.294	-2.94 (-5.34, -0.54)	0.016
PCA SES Component 2 (linear splines)						
Spline 1 (z-score < -1)	0.17 (-3.02, 3.36)	0.918	4.15 (-0.63, 8.93)	0.089	7.84 (-3.27, 12.40)	0.001
Spline 2 (z-score -1 to 0)	0.99 (-1.76, 3.75)	0.480	1.25 (-3.32, 5.82)	0.594	-2.19 (-9.54, 5.15)	0.628
Spline 3 (z-score >0 to 1)	-0.38 (-3.63, 2.88)	0.820	0.44 (-4.28, 5.17)	0.854	-10.13 (-18.53, -1.72)	0.018
Spline 4 (z-score >1)	-0.37 (-1.69, 0.95)	0.582	0.87 (-0.51, 2.25)	0.217	6.34 (3.28, 9.41)	<0.001
**FEMALES**						
PCA SES Component 1 (z-score)	-0.27 (-1.09, 0.55)	0.526	1.07 (-0.19, 2.33)	0.096	-1.42 (-3.47, 0.62)	0.171
PCA SES Component 2 (linear splines)						
Spline 1 (z-score < -1)	1.89 (0.37, 3.41)	0.015	-1.31 (-3.86, 1.25)	0.316	-2.60 (-9.59, 4.38)	0.465
Spline 2 (z-score -1 to 0)	0.99 (-0.06, 2.03)	0.065	7.16 (3.56, 10.76)	<0.001	3.86 (-3.54, 11.26)	0.306
Spline 3 (z-score 0 to 1)	0.52 (-0.99, 2.04)	0.498	-5.91 (-8.32, -3.49)	<0.001	-2.31 (-932, 4.71)	0.519
Spline 4 (z-score >1)	0.07 (-1.99, 2.14)	0.944	-0.73 (-1.86, 0.41)	0.209	3.05 (0.20, 5.90)	0.036

PCA = Principal Components Analysis; SES = socioeconomic status; JHLS-II Jamaica Health and Lifestyle Survey 2007-2008

Models adjusted for age, household SES, study, BMI z-score, glucose, physical activity, and fast food consumption

P-values from three level linear mixed effect models in Stata. Level 1 – individual study participants; level 2 – communities in which participant lived; level 3 – parish in which community was situated. Models used multiple imputation to account for missing data and used robust standard errors.

Table S12: Multivariable models for association between diastolic blood pressure and neighbourhood socioeconomic status for male and female participants in each study

Characteristics	1986 Birth Cohort		Youth Risk Survey		JHLS-II	
**MALES**	**β (95% CI)**	**p-value**	**β (95% CI)**	**p-value**	**β (95% CI)**	**p-value**
PCA SES Component 1 (z-score)	-0.04 (-1.21, 1.13)	0.945	1.10 (0.13, 2.07)	0.026	-0.90 (-3.28, 1.47)	0.456
PCA SES Component 2 (linear splines)						
Spline 1 (z-score < -1)	-5.58 (-6.64, -4.52)	<0.001	-3.86 (-13.51, 5.79)	0.433	4.63 (-3.17, 12.43)	0.245
Spline 2 (z-score -1 to 0)	3.85 (0.48, 7.22)	0.025	3.76 (-0.21, 7.73)	0.063	-2.0 (-10.94, 6.94)	0.662
Spline 3 (z-score >0 to 1)	0.60 (-3.28, 4.48)	0.761	-7.37 (-10.21, -4.53)	<0.001	0.67 (-8.88, 10.22)	0.890
Spline 4 (z-score >1)	-1.03 (-1.90, -0.16)	0.021	2.95 (2.39, 3.51)	<0.001	-4.20 (-6.68, -1.73)	0.001
**FEMALES**						
PCA SES Component 1 (z-score)	1.12 (0.68, 1.55)	<0.001	1.33 (-0.06, 2.72)	0.060	0.50 (-1.29, 2.29)	0.581
PCA SES Component 2 (linear splines)						
Spline 1 (z-score < -1)	1.92 (0.80, 3.04)	0.001	-1.61 (-6.21, 2.99)	0.492	1.95 (-5.62, 9.53)	0.613
Spline 2 (z-score -1 to 0)	-0.43 (-2.14, 1.28)	0.621	3.54 (-0.91, 7.98)	0.119	-2.68 (-9.80, 4.44)	0.461
Spline 3 (z-score 0 to 1)	2.73 1.62, 3.83)	<0.001	-8.05 (-11.74, -4.37)	<0.001	0.99 (-4.90, 6.88)	0.741
Spline 4 (z-score >1)	-0.69 (-1.22, -0.15)	0.013	1.25 (0.20, 2.31)	0.020	0.42 (-1.55, 2.38)	0.676

PCA = Principal Components Analysis; SES = socioeconomic status; JHLS-II Jamaica Health and Lifestyle Survey 2007-2008

Models adjusted for age, household SES, study, BMI z-score, glucose, physical activity, and fast food consumption

P-values from three level linear mixed effect models in Stata. Level 1 – individual study participants; level 2 – communities in which participant lived; level 3 – parish in which community was situated. Models used multiple imputation to account for missing data and used robust standard errors.

Table S13: Full multivariable model for association between systolic blood pressure and covariates for male and female participants

Characteristics	Males β-coefficient (95% CI)		Females β-coefficient (95% CI)	
PCA SES Component 1 (z-score)	-1.48 (-2.11, -0.84)	<0.001	-0.48 (-1.62, 0.66)	0.410
PCA SES Component 2 (linear splines)				
Spline 1 (z-score < -1)	0.10 (-4.01, 3.82)	0.962	-0.43 (-2.74, 1.89)	0.719
Spline 2 (z-score -1 to 0)	1.06 (-2.41, 4.52)	0.550	4.09 (1.49, 6.69)	0.002
Spline 3 (z-score >0 to 1)	-0.39 (-3.25, 2.48)	0.790	-2.81 (-5.04, -0.59)	0.013
Spline 4 (z-score >1)	0.76 (-0.21, 1.73)	0.127	0.63 (-1.05, 2.31)	0.463
Age (years)	0.62 (-0.02, 1.26)	0.059	0.27 (-0.12, 0.65)	0.176
Household Possessions Category				
Lower Third	Reference category		Reference category	
Middle Third	-1.31 (-2.27, -0.36)	0.007	-0.55 (-1.26, 0.17)	0.137
Upper Third	-0.89 (-2.98, 1.19)	0.402	-1.42 (-2.27, -0.56)	0.001
Study				
1986 Birth Cohort	Reference category		Reference category	
Youth Risk Survey	-0.02 (-4.82, 4.77)	0.991	2.14 (0.47, 3.80)	0.012
JHLS-II	2.55 (-0.88, 5.98)	0.144	3.06 (2.01, 4.12)	<0.001
Fasting glucose (mmol/l)	1.03 (-0.15, 2.22)	0.088	0.45 (-0.06, 0.96)	0.087
Body mass index (z-score)	2.74 (1.93, 3.54)	<0.001	1.75 (1.37, 2.14)	<0.001
Physical Activity				
High	Reference category		Reference category	
Moderate	-0.36 (-2.20, 1.49)	0.705	0.54 (-1.30, 2.38)	0.566
Low	-1.39 (-3.12, 0.34)	0.116	0.06 (-1.34, 1.47)	0.931
Fast Food consumption				
<= 2 times/week	Reference category		Reference category	
3-4 times/week	0.60 (-0.86, 2.06)	0.420	-0.60 (-1.42, 0.22)	0.152
>= 5 times/week	-0.80 (-2.90, 1.29)	0.452	-0.12 (-1.39, 1.15)	0.854

PCA = Principal Components Analysis; SES = socioeconomic status

Model adjusted for age, household SES, study, BMI z-score, glucose, physical activity, and fast food consumption

P-values from three level linear mixed effect models in Stata. Level 1 – individual study participants; level 2 – communities in which participant lived; level 3 – parish in which community was situated. Models used multiple imputation to account for missing data and used robust standard errors.

Table S14: Full multivariable model for association between diastolic blood pressure and covariates for male and female participants

Characteristics	Males β-coefficient (95% CI)		Females β-coefficient (95% CI)	
PCA SES Component 1 (z-score)	-0.13 (-0.84, 0.59)	0.730	0.50 (-0.65, 1.65)	0.395
PCA SES Component 2 (linear splines)				
Spline 1 (z-score < -1)	-4.10 (-6.11, -2.09)	<0.001	1.06 (0.15, 1.98)	0.023
Spline 2 (z-score -1 to 0)	2.85 (1.05, 4.66)	0.002	0.27 (-1.58, 2.11)	0.777
Spline 3 (z-score >0 to 1)	-2.36 (-4.88, 0.17)	0.067	-1.49 (-3.73, 0.76)	0.194
Spline 4 (z-score >1)	0.60 (-0.25, 1.44)	0.167	0.46 (-0.61, 1.54)	0.397
Age (years)	0.68 (0.24, 1.11)	0.002	0.18 (-0.02, 0.38)	0.076
Household Possessions Category				
Lower Third	Reference category		Reference category	
Middle Third	-0.75 (-2.14, 0.64)	0.292	0.16 (-0.65, 0.96)	0.706
Upper Third	-0.99 (-2.36, 0.37)	0.155	-0.69 (-1.93, 0.54)	0.271
Study				
1986 Birth Cohort	Reference category		Reference category	
Youth Risk Survey	2.38 (-1.70, 6.45)	0.253	3.22 (1.92, 4.52)	<0.001
JHLS-II	2.48 (0.84, 4.12)	0.003	3.49 (1.41, 5.57)	0.001
Fasting glucose (mmol/l)	0.37 (-0.63, 0.1.37)	0.471	0.50 (0.03, 0.97)	0.037
Body mass index (z-score)	0.45 (-0.63, 1.53)	0.416	0.71 (0.23, 1.18)	0.004
Physical Activity				
High	Reference category		Reference category	
Moderate	0.63 (-1.49, 2.75)	0.560	0.41 (-0.26, 1.09)	0.227
Low	-0.75 (-2.62, 1.11)	0.428	-0.71 (-1.52, 0.10)	0.086
Fast Food consumption				
<= 2 times/week	Reference category		Reference category	
3-4 times/week	0.53 (-1.15, 2.21)	0.537	0.26 (-0.87, 1.40)	0.651
>= 5 times/week	1.24 (-0.69, 3.16)	0.208	-1.00 (-2.38, 0.37)	0.152

PCA = Principal Components Analysis; SES = socioeconomic status

Model adjusted for age, household SES, study, BMI z-score, glucose, physical activity, and fast food consumption

P-values from three level linear mixed effect models in Stata. Level 1 – individual study participants; level 2 – communities in which participant lived; level 3 – parish in which community was situated. Models used multiple imputation to account for missing data and used robust standard errors.

Table S15: Full multivariable model for association between elevated blood pressure or hypertension and covariates for male and female participants

Characteristics	Males Odds ratio (95% CI)		Females Odds ratio (95% CI)	
PCA SES Component 1 (Thirds)				
Lower Third	Reference category		Reference category	
Middle Third	0.81 (0.58, 1.13)	0.201	0.94 (0.65, 1.37)	0.742
Upper Third	0.67 (0.44, 1.00)	0.051	1.08 (0.67, 1.74)	0.760
PCA SES Component 1 (Thirds)				
Lower Third	Reference category		Reference category	
Middle Third	1.02 (0.70, 1.49)	0.910	1.21 (0.79, 1.86)	0.387
Upper Third	0.85 (0.59, 1.24)	0.400	1.10 (0.70, 1.74)	0.684
Age categories				
15-17 years	Reference category		Reference category	
18-19 years	0.97 (0.66, 1.43)	0.891	1.39 (0.97, 2.0)	0.076
20-24 year	1.10 (0.62, 1.94)	0.743	1.55 (0.92, 2.62)	0.101
Household Possessions Category				
Lower Third	Reference category		Reference category	
Middle Third	0.94 (0.68, 1.42)	0.680	1.11 (0.81, 1.51)	0.515
Upper Third	0.88 (0.62, 1.24)	0.451	0.67 (0.46, 0.97)	0.033
Study				
1986 Birth Cohort	Reference category		Reference category	
Youth Risk Survey	1.08 (0.68, 1.71)	0.749	3.06 (1.88, 4.99)	<0.001
JHLS-II	1.50 (0.90, 2.50)	0.123	2.84 (1.68, 4.79)	<0.001
Body mass index categories				
Underweight	0.79 (0.51, 1.25)	0.316	0.80 (0.52, 1.23)	0.307
Normal weight	Reference category		Reference category	
Overweight	1.37 (0.91, 2.07)	0.135	1.72 (1.22, 2.43)	0.002
Obesity	4.97 (2.75, 8.98)	<0.001	2.67 (1.75, 4.06)	<0.001
High glucose (upper quintile)	1.76 (1.26, 2.45)	0.001	1.19 (0.80, 1.76)	0.386
Physical Activity				
High	Reference category		Reference category	
Moderate	0.64 (0.46, 0.88)	0.007	1.56 (1.07, 2.29)	0.022
Low	0.66 (0.47, 0.94)	0.021	1.07 (0.75, 1.52)	0.718
Fast Food consumption				
<= 2 times/week	Reference category		Reference category	
3-4 times/week	1.00 (0.65.1.54)	0.996	0.83 (0.54, 1.27)	0.386
>= 5 times/week	1.06 (0.62, 1.80)	0.841	0.70 (0.40, 1.24)	0.219

PCA = Principal Components Analysis; SES = socioeconomic status

Analyses included 1108 males and 1438 females

Model adjusted for age, household SES, study, BMI z-score, glucose, physical activity, and fast food consumption

P-values from three level logistic mixed effect models in Stata. Level 1 – individual study participants; level 2 – communities in which participant lived; level 3 – parish in which community was situated. Models used multiple imputation to account for missing data.

Figures

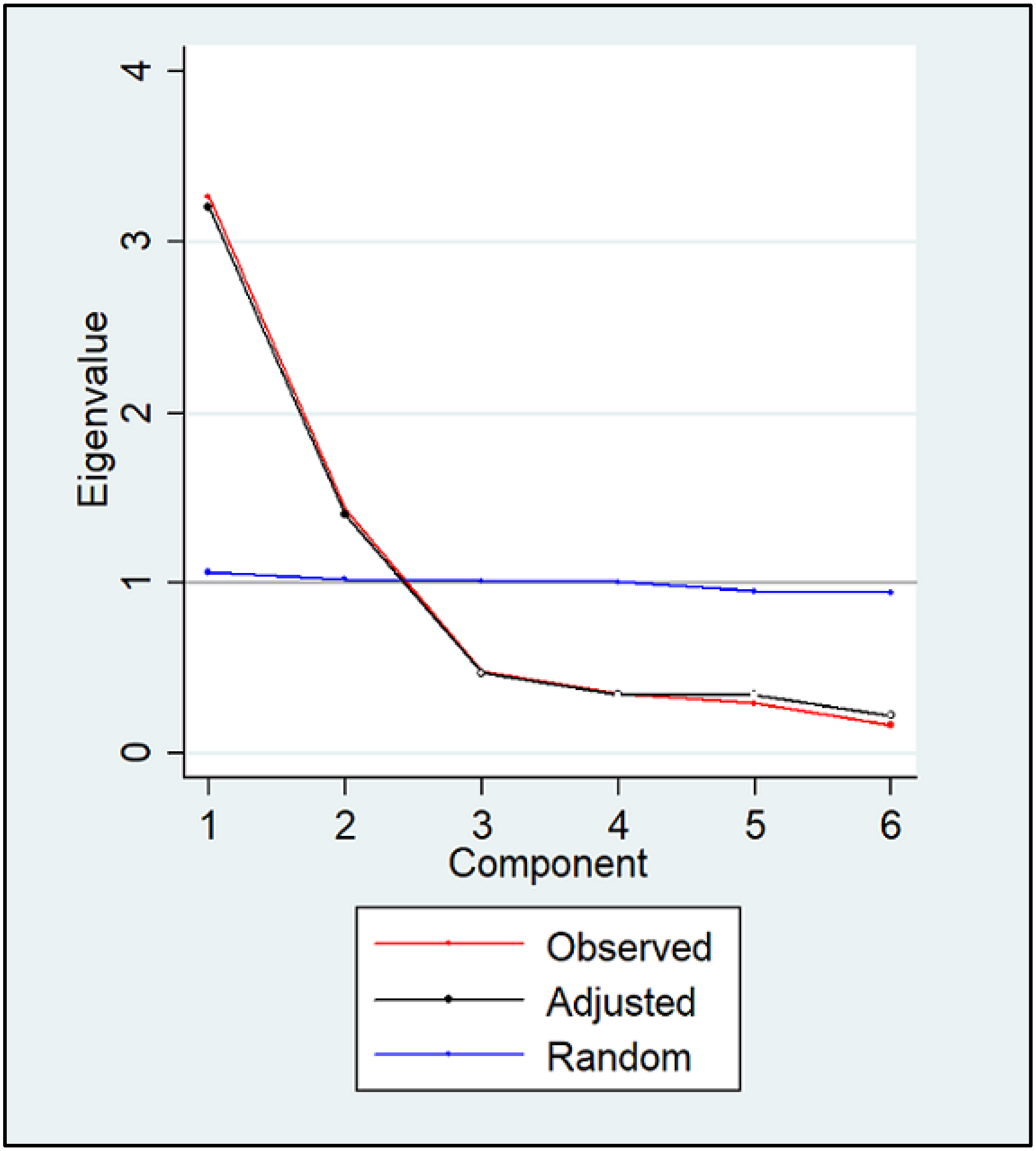

Figure S1: Graph showing adjusted and observed eigenvalues for the components produced by PCA using Horn’s parallel analysis method

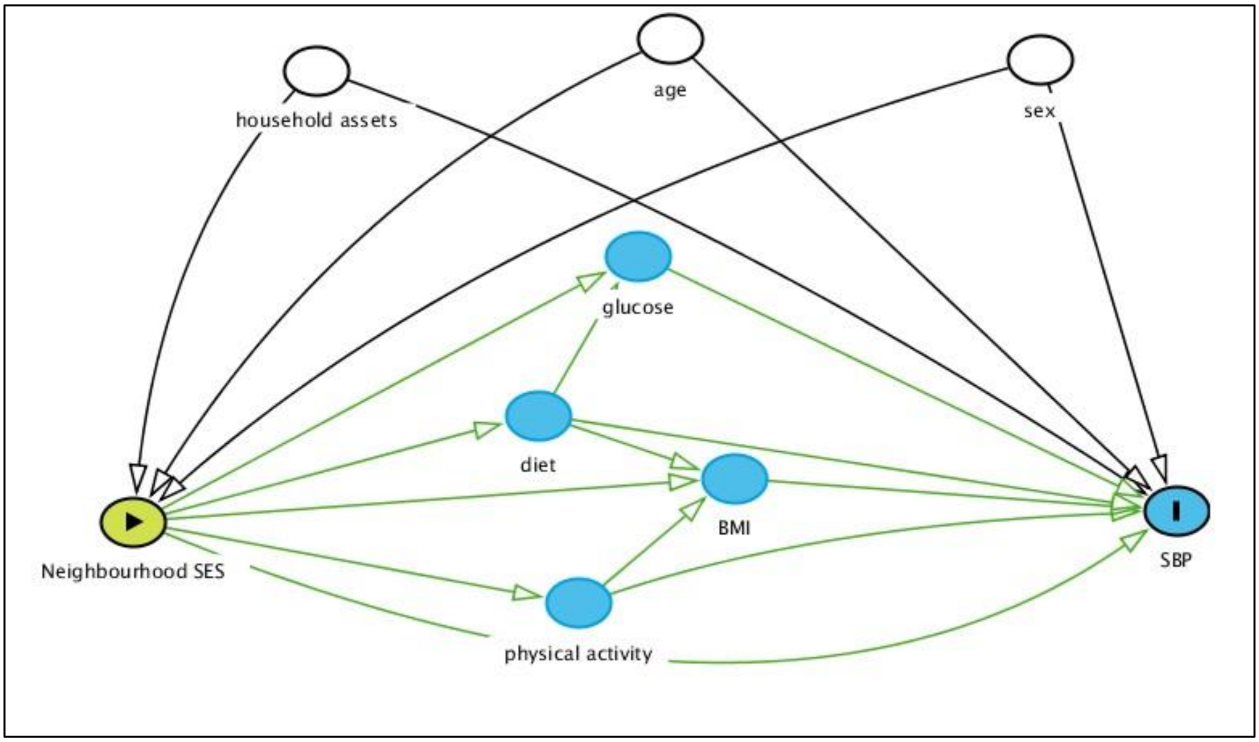

Figure S2: Directed acyclic graph illustrating the hypothesized relationships between blood pressure, neighbourhood socioeconomic status, and covariates

SBP = systolic blood pressure; SES = socioeconomic status
