# Supplementary material for: Neighbourhood socioeconomic characteristics and blood pressure among Jamaican youth: a pooled analysis of data from observational studies
Source: PeerJ. 2020 Oct 6;8:e10058. doi: 10.7717/peerj.10058 (PMC7546221; doi:10.7717/peerj.10058)
Supplement: Supplemental Information 2 [file peerj-08-10058-s002.docx]

**Syntax guide for imputation and analysis commands for Neighbourhood SES and BP paper (revision1 2020-04-21)**

**Commands for Imputation modified 2020-02-27**

****VARIABLES TO BE IMPUTED*****

***Obtaing number of missing values for variables

misstable summarize if include==1

misstable summarize age weight height_cm bmi bmi_std bmicatn4 waistcirc fastglu fast_chol possession_cat3 fastfood_cat2020 phys_act2020 if include==1

**Maximum number of missing values = 152 for fasting cholesterol = approx 6% of total

***Complete case analysis includes 2356 persons = 8% missing data

***27th Feb 2020 - 20 imputations will be done. See Notes file for explanation.

****GENERATING MISSING INDICATOR VARIABLES FOR VARIABLES TO BE IMPUTED*************

gen miss_imputed = missing(age, bmi, height_cm, fastglu, fast_chol, possession_cat3)

tab miss_imputed if include1==1

****COMPARING COMPLETE CASES VS PARTICIPANTS WITH ONE OR MORE MISSING VALUES (29/12/2017)

set more off

for var age weight height_cm bmi mn23sbp mn23dbp fastglu fast_chol highbp_120_80 bmicatn4 ///

populationdensity unemployment poverty murder_rate averagetertiaryeducation averagesecondaryeducation ///

averageprimaryeducation of2bedroomdwellings averagetertiaryeducation_rev pca_comp1 pca_comp2: ///

ttest X if include1==1 , by(miss_imputed) unequal

for var sex bmicatn4 urban_n pca_comp1_tert pca_comp2_tert : ///

tab X miss_imputed if include1==1, col chi

***SAVING MI DATA SETS****2018-02-17

***SAVING MI DATA SETS****2020-02-27

*Test with one imputation

set more off

mi set flong

mi stset, clear

mi register imputed age bmi_std height_cm fastglu fast_chol possession_cat3 fastfood_cat2020 phys_act2020

mi register regular mn23sbp mn23dbp sex pca_comp1_std pca_comp2_std pca_comp2_stdm1 pca_comp2_std0 pca_comp2_std1 pca_comp2_std4

mi impute chained ///

(regress) age bmi_std height_cm fastglu fast_chol ///

(ologit) possession_cat3 fastfood_cat2020 phys_act2020 ///

= mn23sbp mn23dbp sex pca_comp1_std pca_comp2_std if include1== 1, add(1) augment savetrace("trace_2020_02_27", replace) rseed(1234)

count

d, short

***Saving MI data set with 20 imputations

set more off

mi set flong

mi stset, clear

mi register imputed age bmi_std height_cm fastglu fast_chol possession_cat3 fastfood_cat2020 phys_act2020

mi register regular mn23sbp mn23dbp sex pca_comp1_std pca_comp2_std pca_comp2_stdm1 pca_comp2_std0 pca_comp2_std1 pca_comp2_std4

mi impute chained ///

(regress) age bmi_std height_cm fastglu fast_chol ///

(ologit) possession_cat3 fastfood_cat2020 phys_act2020 ///

= mn23sbp mn23dbp sex pca_comp1_std pca_comp2_std if include1== 1, add(20) augment savetrace("trace_2020_02_27_2", replace) rseed(1234)

count

d, short

***Checking Imputed Values***

set more off

for var age bmi height_cm fastglu fast_chol: ///

tabstat X if _mi_m==0 & include1==1, s(n mean sd min max) \ tabstat X if miss_X==1 & include1==1, s(n mean sd min max)

for var possession_cat3: ///

tab X if miss_X==0 & _mi_m==0 & include1==1 \ tab X if miss_X==1 & include1==1

****EXTRACTING IMPUTED DATASET M1 FOR MODEL SELECTION

mi extract 1

count

d, short

**Commands for Analyses**

* List of variables for descriptive tables (updated 2020-03-10)

*Continous variables: age weight height_cm bmi mn23sbp mn23dbp waistcirc fastglu fast_chol pca_comp1_std pca_comp2_std

*Categorical variables: sex possession_cat3 phys_act2020 fastfood_cat2020 bmicatn4 highbp_120_80

*Community variables categorical: urban_n pca_comp1_tert pca_comp2_tert

*Community variables: continuous: population populationdensity unemployment poverty murder_rate ///

*averagetertiaryeducation averagesecondaryeducation averageprimaryeducation of2bedroomdwellings dependencyratio pca_comp1 pca_comp1_tert

***Number of missing values for variables included in analyses

misstable sum age weight height_cm bmi mn23sbp mn23dbp waistcirc fastglu fast_chol pca_comp1_std pca_comp2_std ///

sex possession_cat3 phys_act2020 fastfood_cat2020 bmicatn4 highbp_120_80 ///

if include1==1

egen num_missing = rowmiss(age weight height_cm bmi mn23sbp mn23dbp waistcirc fastglu fast_chol pca_comp1_std pca_comp2_std ///

sex possession_cat3 phys_act2020 fastfood_cat2020 bmicatn4 highbp_120_80) ///

if include1==1

****COMMANDS FOR TABLE 1 - INDIVIDUAL PARTICIPANT and COMMUNITY CONTINUOUS DESCRIPTIVE DATA (Modified 2020-03-10)

set more off

for var age weight height_cm bmi mn23sbp mn23dbp waistcirc fastglu fast_chol pca_comp1_std pca_comp2_std: ///

sum X if include1==1 \ oneway X study if include1==1, tabulate \ kwallis X if include1==1, by(study)

set more off

for var sex highbp_120_80 possession_cat3 phys_act2020 fastfood_cat2020 bmicatn4 : ///

tab X study if include1==1, col chi

****COMMANDS FOR TABLE 1 SUPPLEMENT / APPENDIX - INDIVIDUAL PARTICIPANT CONTINUOUS DESCRIPTIVE DATA (Modified 2020-03-10)

set more off

for var age weight height_cm bmi mn23sbp mn23dbp waistcirc fastglu fast_chol pca_comp1_std pca_comp2_std: ///

bysort study: ttest X if include1==1, by(sex) unequal

set more off

for var sex highbp_120_80 possession_cat3 phys_act2020 fastfood_cat2020 bmicatn4 : ///

bysort study: tab X sex if include1==1, col chi

****COMMANDS FOR TABLE 2 - INDIVIDUAL PARTICIPANT and COMMUNITY CONTINUOUS DESCRIPTIVE DATA (Modified 2020-03-21)

set more off

for var age weight height_cm bmi mn23sbp mn23dbp waistcirc fastglu fast_chol pca_comp1_std pca_comp2_std: ///

ttest X if include1==1, by(highbp_120_80) unequal

set more off

for var sex possession_cat3 phys_act2020 fastfood_cat2020 bmicatn4 : ///

tab X highbp_120_80 if include1==1, col chi

****COMMANDS FOR TABLE 3 - MULTILEVL MIXED EFFECTS UNIVARIATE REGRESSION models (Modified 2020-04-14)

***Robust standard errors for SBP****

***SBP

**MALES

set more off

for var age bmi_std fastglu pca_comp1_std pca_comp2_std: ///

mi estimate: mixed mn23sbp X if include1==1 & sex==1 || parish: || community_n:, vce(robust)

set more off

for var pca_comp2_stdm1 pca_comp2_std0 pca_comp2_std1 pca_comp2_std4: ///

mi estimate: mixed mn23sbp X if include1==1 & sex==1 || parish: || community_n:, vce(robust)

for var possession_cat3 phys_act2020 fastfood_cat2020 : ///

mi estimate: mixed mn23sbp i.X if include1==1 & sex==1 || parish: || community_n:, vce(robust)

***SBP

**FEMALES

set more off

for var age bmi_std fastglu pca_comp1_std pca_comp2_std: ///

mi estimate: mixed mn23sbp X if include1==1 & sex==0 || parish: || community_n:, vce(robust)

set more off

for var pca_comp2_stdm1 pca_comp2_std0 pca_comp2_std1 pca_comp2_std4: ///

mi estimate: mixed mn23sbp X if include1==1 & sex==0 || parish: || community_n:, vce(robust)

for var possession_cat3 phys_act2020 fastfood_cat2020 : ///

mi estimate: mixed mn23sbp i.X if include1==1 & sex==0 || parish: || community_n:, vce(robust)

***DBP

**MALES

set more off

for var age bmi_std fastglu pca_comp1_std pca_comp2_std: ///

mi estimate: mixed mn23dbp X if include1==1 & sex==1 || parish: || community_n: , vce(robust)

set more off

for var pca_comp2_stdm1 pca_comp2_std0 pca_comp2_std1 pca_comp2_std4: ///

mi estimate: mixed mn23dbp X if include1==1 & sex==1 || parish: || community_n: , vce(robust)

for var possession_cat3 phys_act2020 fastfood_cat2020 : ///

mi estimate: mixed mn23dbp i.X if include1==1 & sex==1 || parish: || community_n:, vce(robust)

***DBP

**FEMALES

set more off

for var age bmi_std fastglu pca_comp1_std pca_comp2_std: ///

mi estimate: mixed mn23dbp X if include1==1 & sex==0 || parish: || community_n:, vce(robust)

set more off

for var pca_comp2_stdm1 pca_comp2_std0 pca_comp2_std1 pca_comp2_std4: ///

mi estimate: mixed mn23dbp X if include1==1 & sex==0 || parish: || community_n:, vce(robust)

for var possession_cat3 phys_act2020 fastfood_cat2020 : ///

mi estimate: mixed mn23dbp i.X if include1==1 & sex==0 || parish: || community_n:, vce(robust)

****Generating Categorical variables to use in multilevel logistic regression models*****March 26, 2020

set more off

mi passive: gen agecat = .

mi passive: replace agecat = 1 if age <18 & include1==1

mi passive: replace agecat = 2 if age >=18 & age <20 & include1==1

mi passive: replace agecat = 3 if age >=20 & age !=. & include1==1

set more off

mi passive: gen fastglu_quin5 = .

mi passive: replace fastglu_quin5 =0 if fastglu < 4.7924 & include1==1

mi passive: replace fastglu_quin5 =1 if fastglu > 4.7924 & fastglu !=. & include1==1

****COMMANDS FOR Appendix TABLE X - MULTILEVL Logistic regression UNIVARIATE models using categorical dependent variables (Modified 2020-03-26)

***Do file name "Syntax_guide_2018_03_05_1 analysis commands (revision1 2020-03-10) correct"

**MALES

set more off

for var agecat bmicatn4 fastglu_quin5 pca_comp1_tert pca_comp2_tert possession_cat3 phys_act2020 fastfood_cat2020 : ///

mi estimate, or: meqrlogit highbp_120_80 i.X if include1==1 & sex==1 || parish: || community_n:

**FEMALES

set more off

for var agecat bmicatn4 fastglu_quin5 pca_comp1_tert pca_comp2_tert possession_cat3 phys_act2020 fastfood_cat2020 : ///

mi estimate, or: meqrlogit highbp_120_80 i.X if include1==1 & sex==0 || parish: || community_n:

****New FINAL MMODELS****2020-04-14

*****Robust standard errors

***Do file name "Syntax_guide_2018_03_05_1 analysis commands (revision1 2020-04-14) correct"

*Sex-specific sequential model

****SBP

set more off

***Males

***Model 1

mi estimate: mixed mn23sbp pca_comp1_std pca_comp2_stdm1 pca_comp2_std0 pca_comp2_std1 pca_comp2_std4 age i.possession_cat3 ///

i.study if include1==1 & sex==1 || parish: || community_n: , vce(robust)

***Model 2

mi estimate: mixed mn23sbp pca_comp1_std pca_comp2_stdm1 pca_comp2_std0 pca_comp2_std1 pca_comp2_std4 age i.possession_cat3 ///

i.study bmi_std if include1==1 & sex==1 || parish: || community_n: , vce(robust)

****Model 3

mi estimate: mixed mn23sbp pca_comp1_std pca_comp2_stdm1 pca_comp2_std0 pca_comp2_std1 pca_comp2_std4 age i.possession_cat3 ///

i.study fastglu bmi_std i.phys_act2020 i.fastfood_cat2020 if include1==1 & sex==1 || parish: || community_n:, vce(robust)

***Females

set more off

***Model 1

mi estimate: mixed mn23sbp pca_comp1_std pca_comp2_stdm1 pca_comp2_std0 pca_comp2_std1 pca_comp2_std4 age i.possession_cat3 ///

i.study if include1==1 & sex==0 || parish: || community_n: , vce(robust)

***Model 2

mi estimate: mixed mn23sbp pca_comp1_std pca_comp2_stdm1 pca_comp2_std0 pca_comp2_std1 pca_comp2_std4 age i.possession_cat3 ///

i.study bmi_std if include1==1 & sex==0 || parish: || community_n: , vce(robust)

****Model 3

mi estimate: mixed mn23sbp pca_comp1_std pca_comp2_stdm1 pca_comp2_std0 pca_comp2_std1 pca_comp2_std4 age i.possession_cat3 ///

i.study fastglu bmi_std i.phys_act2020 i.fastfood_cat2020 if include1==1 & sex==0 || parish: || community_n:, vce(robust)

*Sex-specific sequential model

****DBP

set more off

***Males

***Model 1

mi estimate: mixed mn23dbp pca_comp1_std pca_comp2_stdm1 pca_comp2_std0 pca_comp2_std1 pca_comp2_std4 age i.possession_cat3 ///

i.study if include1==1 & sex==1 || parish: || community_n: , vce(robust)

***Model 2

mi estimate: mixed mn23dbp pca_comp1_std pca_comp2_stdm1 pca_comp2_std0 pca_comp2_std1 pca_comp2_std4 age i.possession_cat3 ///

i.study bmi_std if include1==1 & sex==1 || parish: || community_n: , vce(robust)

****Model 3

mi estimate: mixed mn23dbp pca_comp1_std pca_comp2_stdm1 pca_comp2_std0 pca_comp2_std1 pca_comp2_std4 age i.possession_cat3 ///

i.study fastglu bmi_std i.phys_act2020 i.fastfood_cat2020 if include1==1 & sex==1 || parish: || community_n:, vce(robust)

***Females

set more off

***Model 1

mi estimate: mixed mn23dbp pca_comp1_std pca_comp2_stdm1 pca_comp2_std0 pca_comp2_std1 pca_comp2_std4 age i.possession_cat3 ///

i.study if include1==1 & sex==0 || parish: || community_n: , vce(robust)

***Model 2

mi estimate: mixed mn23dbp pca_comp1_std pca_comp2_stdm1 pca_comp2_std0 pca_comp2_std1 pca_comp2_std4 age i.possession_cat3 ///

i.study bmi_std if include1==1 & sex==0 || parish: || community_n: , vce(robust)

****Model 3

mi estimate: mixed mn23dbp pca_comp1_std pca_comp2_stdm1 pca_comp2_std0 pca_comp2_std1 pca_comp2_std4 age i.possession_cat3 ///

i.study fastglu bmi_std i.phys_act2020 i.fastfood_cat2020 if include1==1 & sex==0 || parish: || community_n: , vce(robust)

*Sex-specific sequential model

****Elevated BP/HTN

*agecat bmicatn4 fastglu_quin5 highbp_120_80 pca_comp1_tert pca_comp2_tert agecat

set more off

***Males

***Model 1

mi estimate, or: meqrlogit highbp_120_80 i.pca_comp1_tert i.pca_comp2_tert i.agecat i.possession_cat3 ///

i.study if include1==1 & sex==1 || parish: || community_n:

***Model 2

mi estimate, or: meqrlogit highbp_120_80 i.pca_comp1_tert i.pca_comp2_tert i.agecat i.possession_cat3 ///

i.study i.bmicatn4 if include1==1 & sex==1 || parish: || community_n:

****Model 3

mi estimate, or: meqrlogit highbp_120_80 i.pca_comp1_tert i.pca_comp2_tert i.agecat i.possession_cat3 ///

i.study i.bmicatn4 i.fastglu_quin5 i.phys_act2020 i.fastfood_cat2020 if include1==1 & sex==1 || parish: || community_n:

***Females

set more off

***Model 1

mi estimate, or: meqrlogit highbp_120_80 i.pca_comp1_tert i.pca_comp2_tert i.agecat i.possession_cat3 ///

i.study if include1==1 & sex==0 || parish: || community_n:

***Model 2

mi estimate, or: meqrlogit highbp_120_80 i.pca_comp1_tert i.pca_comp2_tert i.agecat i.possession_cat3 ///

i.study i.bmicatn4 if include1==1 & sex==0 || parish: || community_n:

****Model 3

mi estimate, or: meqrlogit highbp_120_80 i.pca_comp1_tert i.pca_comp2_tert i.agecat i.possession_cat3 ///

i.study i.bmicatn4 i.fastglu_quin5 i.phys_act2020 i.fastfood_cat2020 if include1==1 & sex==0 || parish: || community_n:

***RERUN of MODEL 3 for SBP and DBP for Individual Studies*****

***Do file name "Syntax_guide_2018_03_05_1 analysis commands (revision1 2020-03-10) correct"

***MALES

***SBP

****Model 3

set more off

***1986 Birth Cohort

mi estimate: mixed mn23sbp pca_comp1_std pca_comp2_stdm1 pca_comp2_std0 pca_comp2_std1 pca_comp2_std4 age i.possession_cat3 ///

fastglu bmi_std i.phys_act2020 i.fastfood_cat2020 if include1==1 & sex==1 & study ==1 || parish: || community_n:, vce(robust)

***Youth Risk

mi estimate: mixed mn23sbp pca_comp1_std pca_comp2_stdm1 pca_comp2_std0 pca_comp2_std1 pca_comp2_std4 age i.possession_cat3 ///

fastglu bmi_std i.phys_act2020 i.fastfood_cat2020 if include1==1 & sex==1 & study ==2 || parish: || community_n:, vce(robust)

***JHLS-II

mi estimate: mixed mn23sbp pca_comp1_std pca_comp2_stdm1 pca_comp2_std0 pca_comp2_std1 pca_comp2_std4 age i.possession_cat3 ///

fastglu bmi_std i.phys_act2020 i.fastfood_cat2020 if include1==1 & sex==1 & study ==3 || parish: || community_n:, vce(robust)

***DBP

****Model 3

set more off

***1986 Birth Cohort

mi estimate: mixed mn23dbp pca_comp1_std pca_comp2_stdm1 pca_comp2_std0 pca_comp2_std1 pca_comp2_std4 age i.possession_cat3 ///

fastglu bmi_std i.phys_act2020 i.fastfood_cat2020 if include1==1 & sex==1 & study ==1 || parish: || community_n: , vce(robust)

***Youth Risk

mi estimate: mixed mn23dbp pca_comp1_std pca_comp2_stdm1 pca_comp2_std0 pca_comp2_std1 pca_comp2_std4 age i.possession_cat3 ///

fastglu bmi_std i.phys_act2020 i.fastfood_cat2020 if include1==1 & sex==1 & study ==2 || parish: || community_n: , vce(robust)

***JHLS-II

mi estimate: mixed mn23dbp pca_comp1_std pca_comp2_stdm1 pca_comp2_std0 pca_comp2_std1 pca_comp2_std4 age i.possession_cat3 ///

fastglu bmi_std i.phys_act2020 i.fastfood_cat2020 if include1==1 & sex==1 & study ==3 || parish: || community_n: , vce(robust)

***FEMALES

***SBP

****Model 3

set more off

***1986 Birth Cohort

mi estimate: mixed mn23sbp pca_comp1_std pca_comp2_stdm1 pca_comp2_std0 pca_comp2_std1 pca_comp2_std4 age i.possession_cat3 ///

fastglu bmi_std i.phys_act2020 i.fastfood_cat2020 if include1==1 & sex==0 & study ==1 || parish: || community_n:, vce(robust)

***Youth Risk

mi estimate: mixed mn23sbp pca_comp1_std pca_comp2_stdm1 pca_comp2_std0 pca_comp2_std1 pca_comp2_std4 age i.possession_cat3 ///

fastglu bmi_std i.phys_act2020 i.fastfood_cat2020 if include1==1 & sex==0 & study ==2 || parish: || community_n:, vce(robust)

***JHLS-II

mi estimate: mixed mn23sbp pca_comp1_std pca_comp2_stdm1 pca_comp2_std0 pca_comp2_std1 pca_comp2_std4 age i.possession_cat3 ///

fastglu bmi_std i.phys_act2020 i.fastfood_cat2020 if include1==1 & sex==0 & study ==3 || parish: || community_n:, vce(robust)

***DBP

****Model 3

set more off

***1986 Birth Cohort

mi estimate: mixed mn23dbp pca_comp1_std pca_comp2_stdm1 pca_comp2_std0 pca_comp2_std1 pca_comp2_std4 age i.possession_cat3 ///

fastglu bmi_std i.phys_act2020 i.fastfood_cat2020 if include1==1 & sex==0 & study ==1 || parish: || community_n: , vce(robust)

***Youth Risk

mi estimate: mixed mn23dbp pca_comp1_std pca_comp2_stdm1 pca_comp2_std0 pca_comp2_std1 pca_comp2_std4 age i.possession_cat3 ///

fastglu bmi_std i.phys_act2020 i.fastfood_cat2020 if include1==1 & sex==0 & study ==2 || parish: || community_n: , vce(robust)

***JHLS-II

mi estimate: mixed mn23dbp pca_comp1_std pca_comp2_stdm1 pca_comp2_std0 pca_comp2_std1 pca_comp2_std4 age i.possession_cat3 ///

fastglu bmi_std i.phys_act2020 i.fastfood_cat2020 if include1==1 & sex==0 & study ==3 || parish: || community_n: , vce(robust)

****COMPLETE CASE ANALYSIS*****

****Model 3

set more off

****SBP

bysort sex: mixed mn23sbp pca_comp1_std pca_comp2_stdm1 pca_comp2_std0 pca_comp2_std1 pca_comp2_std4 age i.possession_cat3 ///

i.study fastglu bmi_std i.phys_act2020 i.fastfood_cat2020 if include1==1 & _mi_m==0 || parish: || community_n:, vce(robust)

****DBP

bysort sex: mixed mn23dbp pca_comp1_std pca_comp2_stdm1 pca_comp2_std0 pca_comp2_std1 pca_comp2_std4 age i.possession_cat3 ///

i.study fastglu bmi_std i.phys_act2020 i.fastfood_cat2020 if include1==1 & _mi_m==0 || parish: || community_n: , vce(robust)

***MODEL DIAGNOSTICS - modified 2020-04-09

*** DATA SET - IMPUTATION DATA SET, M1, USED

***SBP

***Males

****Model 3

set more off

mixed mn23sbp pca_comp1_std pca_comp2_stdm1 pca_comp2_std0 pca_comp2_std1 pca_comp2_std4 age i.possession_cat3 ///

i.study fastglu bmi_std i.phys_act2020 i.fastfood_cat2020 if include1==1 & sex==1 || parish: || community_n:

predict stdresid_male_nolevel if e(sample) , rstandard

/*mixed mn23sbp pca_comp1_std pca_comp2_stdm1 pca_comp2_std0 pca_comp2_std1 pca_comp2_std4 age i.possession_cat3 ///

i.study fastglu bmi_std i.phys_act2020 i.fastfood_cat2020 if include1==1 & sex==1 & stdresid_male_nolevel >= -2.5 & stdresid_male_nolevel <= 2.5 || parish: || community_n:

*/

mixed mn23sbp pca_comp1_std pca_comp2_stdm1 pca_comp2_std0 pca_comp2_std1 pca_comp2_std4 age i.possession_cat3 ///

i.study fastglu bmi_std i.phys_act2020 i.fastfood_cat2020 if include1==1 & sex==1 & stdresid_male_nolevel >= -3 & stdresid_male_nolevel <= 3 || parish: || community_n:

qnorm stdresid_male_nolevel

qnorm stdresid_male_nolevel if stdresid_male_nolevel >= -3 & stdresid_male_nolevel <= 3

sktest stdresid_male_nolevel

sktest stdresid_male_nolevel if stdresid_male_nolevel >= -3 & stdresid_male_nolevel <= 3

dis 1100/1110 /* Estimating the proportion within the mid-99% range*/

br stdresid_male_nolevel mn23sbp if (stdresid_male_nolevel < -3 | stdresid_male_nolevel > 3 )& stdresid_male_nolevel != .

***Females

set more off

****Model 3

mixed mn23sbp pca_comp1_std pca_comp2_stdm1 pca_comp2_std0 pca_comp2_std1 pca_comp2_std4 age i.possession_cat3 ///

i.study fastglu bmi_std i.phys_act2020 i.fastfood_cat2020 if include1==1 & sex==0 || parish: || community_n:

predict stdresid_female_nolevel if e(sample) , rstandard

/* mixed mn23sbp pca_comp1_std pca_comp2_stdm1 pca_comp2_std0 pca_comp2_std1 pca_comp2_std4 age i.possession_cat3 ///

i.study fastglu bmi_std i.phys_act2020 i.fastfood_cat2020 if include1==1 & sex==0 & stdresid_female_nolevel >= -2.5 & stdresid_female_nolevel <= 2.5 || parish: || community_n:

*/

mixed mn23sbp pca_comp1_std pca_comp2_stdm1 pca_comp2_std0 pca_comp2_std1 pca_comp2_std4 age i.possession_cat3 ///

i.study fastglu bmi_std i.phys_act2020 i.fastfood_cat2020 if include1==1 & sex==0 & stdresid_female_nolevel >= -3 & stdresid_female_nolevel <= 3 || parish: || community_n:

qnorm stdresid_female_nolevel

count if stdresid_female_nolevel >= -3 & stdresid_female_nolevel <= 3 & sex ==0

qnorm stdresid_female_nolevel if stdresid_female_nolevel >= -3 & stdresid_female_nolevel <= 3

br stdresid_female_nolevel mn23sbp if (stdresid_female_nolevel < -3 | stdresid_female_nolevel > 3 )& stdresid_female_nolevel != .

sktest stdresid_female_nolevel if stdresid_female_nolevel >= -3 & stdresid_female_nolevel <= 3

sktest stdresid_female_nolevel

dis 1436/1446 /* Estimating the proportion within the mid-99% range*/

***DBP - reopen on m1 dataset

***Males

****Model 3

set more off

mixed mn23dbp pca_comp1_std pca_comp2_stdm1 pca_comp2_std0 pca_comp2_std1 pca_comp2_std4 age i.possession_cat3 ///

i.study fastglu bmi_std i.phys_act2020 i.fastfood_cat2020 if include1==1 & sex==1 || parish: || community_n:

predict stdresid_male_nolevel if e(sample) , rstandard

/*mixed mn23dbp pca_comp1_std pca_comp2_stdm1 pca_comp2_std0 pca_comp2_std1 pca_comp2_std4 age i.possession_cat3 ///

i.study fastglu bmi_std i.phys_act2020 i.fastfood_cat2020 if include1==1 & sex==1 & stdresid_male_nolevel >= -2.5 & stdresid_male_nolevel <= 2.5 || parish: || community_n:

*/

mixed mn23dbp pca_comp1_std pca_comp2_stdm1 pca_comp2_std0 pca_comp2_std1 pca_comp2_std4 age i.possession_cat3 ///

i.study fastglu bmi_std i.phys_act2020 i.fastfood_cat2020 if include1==1 & sex==1 & stdresid_male_nolevel >= -3 & stdresid_male_nolevel <= 3 || parish: || community_n:

qnorm stdresid_male_nolevel

qnorm stdresid_male_nolevel if stdresid_male_nolevel >= -3 & stdresid_male_nolevel <= 3

sktest stdresid_male_nolevel

sktest stdresid_male_nolevel if stdresid_male_nolevel >= -3 & stdresid_male_nolevel <= 3

dis ... /* Estimating the proportion within the mid-99% range*/

br stdresid_male_nolevel mn23dbp if (stdresid_male_nolevel < -3 | stdresid_male_nolevel > 3 )& stdresid_male_nolevel != .

***Females

set more off

****Model 3

mixed mn23dbp pca_comp1_std pca_comp2_stdm1 pca_comp2_std0 pca_comp2_std1 pca_comp2_std4 age i.possession_cat3 ///

i.study fastglu bmi_std i.phys_act2020 i.fastfood_cat2020 if include1==1 & sex==0 || parish: || community_n:

predict stdresid_female_nolevel if e(sample) , rstandard

/* mixed mn23dbp pca_comp1_std pca_comp2_stdm1 pca_comp2_std0 pca_comp2_std1 pca_comp2_std4 age i.possession_cat3 ///

i.study fastglu bmi_std i.phys_act2020 i.fastfood_cat2020 if include1==1 & sex==0 & stdresid_female_nolevel >= -2.5 & stdresid_female_nolevel <= 2.5 || parish: || community_n:

*/

mixed mn23dbp pca_comp1_std pca_comp2_stdm1 pca_comp2_std0 pca_comp2_std1 pca_comp2_std4 age i.possession_cat3 ///

i.study fastglu bmi_std i.phys_act2020 i.fastfood_cat2020 if include1==1 & sex==0 & stdresid_female_nolevel >= -3 & stdresid_female_nolevel <= 3 || parish: || community_n:

qnorm stdresid_female_nolevel

count if stdresid_female_nolevel >= -3 & stdresid_female_nolevel <= 3 & sex ==0

qnorm stdresid_female_nolevel if stdresid_female_nolevel >= -3 & stdresid_female_nolevel <= 3

br stdresid_female_nolevel mn23dbp if (stdresid_female_nolevel < -3 | stdresid_female_nolevel > 3 )& stdresid_female_nolevel != .

sktest stdresid_female_nolevel if stdresid_female_nolevel >= -3 & stdresid_female_nolevel <= 3

sktest stdresid_female_nolevel

dis ... /* Estimating the proportion within the mid-99% range*/

**Sample Size Calculations**

*Syntax for initial simple random sample size estimation

dis "Sample size calculation for males"

power onecorrelation 0 -0.1363, power(0.8)

*n = 421

dis "Sample size calculation for females"

power onecorrelation 0 -0.1516 , power(0.8)

*n = 340

*Commands below provided sex-specific estimates for ICC

***Relationship between SBP and pca_comp1_std

**MALES

mixed mn23sbp pca_comp1_std if include1==1 & sex==1 || parish: || community_n:

estat icc

*icc = 0.086831

**FEMALES

mixed mn23sbp pca_comp1_std if include1==1 & sex== 0 || parish: || community_n:

estat icc

*icc = 0.1342401

* In order to obtain the average number (m) of persons per cluster (community) we used

bysort sex: xtsum mn23sbp if include1 == 1 , i( community_n)

*Males , m = 5

*Females, m= 6

*After Lohr (1999) (page 240) we estimated the sex-specific design effect values as follows:

dis "Design effect for males = " 1+(5-1)*0.086831

dis "Design effect for females = " 1+(6-1)*0.1342401

* Lohr S. Sampling: Design and Analysis. Pacific Grove: Brooks / Cole Publishing Company; 1999 1999. - Design Effect reference

*The adjusted sample size that will anticipate the effect of clustering in comunities on variance parameter estimates

dis "Sample size adjusted to accommodate effect of clustering - for males = " (1+(5-1)*0.086831)*421

dis "Sample size adjusted to accommodate effect of clustering - for females = " (1+(6-1)*0.1342401)*340
