# Supplementary material for: Neighbourhood socioeconomic characteristics and blood pressure among Jamaican youth: a pooled analysis of data from observational studies
Source: PeerJ. 2020 Oct 6;8:e10058. doi: 10.7717/peerj.10058 (PMC7546221; doi:10.7717/peerj.10058)
Supplement: Supplemental Information 4 [file peerj-08-10058-s004.pdf]

```
--------------------------------------------------------------------------------------------------------------------------------------------
num                                                                                                                            (unlabeled)
--------------------------------------------------------------------------------------------------------------------------------------------

                  type:  numeric (float)

                 range:  [1,2620]                    units:  1
         unique values:  2,620                   missing .:  0/2,620

                  mean:    1310.5
             std. dev:   756.473

           percentiles:        10%       25%       50%       75%       90%
                             262.5     655.5    1310.5    1965.5    2358.5

--------------------------------------------------------------------------------------------------------------------------------------------
sex                                                                                                                                    sex
--------------------------------------------------------------------------------------------------------------------------------------------

                  type:  numeric (float)
                 label:  sex

                 range:  [0,1]                       units:  1
         unique values:  2                       missing .:  0/2,620

           tabulation:  Freq.   Numeric  Label
                        1,485         0  Female
                        1,135         1  Male

--------------------------------------------------------------------------------------------------------------------------------------------
age                                                                                                                  age at last birthday
--------------------------------------------------------------------------------------------------------------------------------------------

                  type:  numeric (float)

                 range:  [15,24]                     units:  1
         unique values:  10                      missing .:  1/2,620

                  mean:   17.9084
             std. dev:   2.04121

           percentiles:        10%       25%       50%       75%       90%
                                15        16        18        19        20

--------------------------------------------------------------------------------------------------------------------------------------------
weight                                                                                                                         weight (kg)
--------------------------------------------------------------------------------------------------------------------------------------------

                  type:  numeric (double)
```

```
                range:  [38.1,170.2]                 units:  .01
        unique values:  878                      missing .:  38/2,620

                 mean:   64.4901
             std. dev:   14.6304

          percentiles:        10%       25%       50%       75%       90%
                             48.6      54.2      62.3      71.4      82.8

------------------------------------------------------------------------------------------------------------------------------------
height_cm                                                                                                                height (cm)
------------------------------------------------------------------------------------------------------------------------------------

                 type:  numeric (double)

                range:  [100.5,208]                  units:  .00001
        unique values:  750                      missing .:  43/2,620

                 mean:   168.002
             std. dev:   9.40494

          percentiles:        10%       25%       50%       75%       90%
                            157.1     161.4    167.55     174.5     180.3

------------------------------------------------------------------------------------------------------------------------------------
bmi                                                                                                                  Body Mass Index
------------------------------------------------------------------------------------------------------------------------------------

                 type:  numeric (float)

                range:  [13.922607,60.667767]        units:  1.000e-06
        unique values:  2,539                    missing .:  45/2,620

                 mean:   22.8652
             std. dev:   5.06445

          percentiles:        10%       25%       50%       75%       90%
                          18.1508   19.5999   21.6171   24.8301   29.0009

------------------------------------------------------------------------------------------------------------------------------------
mn23sbp                                                                                 mean of 2nd and 3rd sytolic blood pressure
------------------------------------------------------------------------------------------------------------------------------------

                 type:  numeric (double)

                range:  [79,170]                     units:  .1
        unique values:  74                       missing .:  63/2,620

                 mean:   111.494
             std. dev:   10.7848
```

```
        percentiles:        10%       25%       50%       75%       90%
                             98       104       111       118       125

------------------------------------------------------------------------------------------------------------------------------------
mn23dbp                                                                                      mean of 2nd and 3rd diastolic blood pressure
------------------------------------------------------------------------------------------------------------------------------------

                type:  numeric (double)

               range:  [0,127]                      units:  .1
       unique values:  78                       missing .:  64/2,620

                mean:   69.8112
            std. dev:    10.186

        percentiles:        10%       25%       50%       75%       90%
                             58        63        70        77        81

------------------------------------------------------------------------------------------------------------------------------------
waistcirc                                                                                                         waist circumference
------------------------------------------------------------------------------------------------------------------------------------

                type:  numeric (float)

               range:  [25.25,183.05]               units:  1.000e-06
       unique values:  1,049                    missing .:  44/2,620

                mean:   73.5961
            std. dev:    10.956

        percentiles:        10%       25%       50%       75%       90%
                             63   66.6167      71.5      77.8   87.4667

------------------------------------------------------------------------------------------------------------------------------------
fastglu                                                                                                        Fasting Plasma glucose
------------------------------------------------------------------------------------------------------------------------------------

                type:  numeric (float)

               range:  [1.2746,28.884001]           units:  1.000e-07
       unique values:  101                      missing .:  190/2,620

                mean:   3.99388
            std. dev:   1.20901

        percentiles:        10%       25%       50%       75%       90%
                         2.5538    3.4066    4.1264    4.6858       5.1
```

```
-------------------------------------------------------------------------------------------------------------------------------------
fast_chol                                                                                                 fasting total cholesterol
-------------------------------------------------------------------------------------------------------------------------------------

                  type:  numeric (double)

                 range:  [0,10]                       units:  .01
         unique values:  169                      missing .:  198/2,620

                  mean:   2.48247
              std. dev:   2.20229

           percentiles:        10%       25%       50%       75%       90%
                                 0         0       3.7      4.29       4.9

-------------------------------------------------------------------------------------------------------------------------------------
highbp_120_80                                                                           elevated blood pressure (>=120/80 mmHg)
-------------------------------------------------------------------------------------------------------------------------------------

                  type:  numeric (float)
                 label:  true

                 range:  [0,1]                        units:  1
         unique values:  2                        missing .:  64/2,620

            tabulation:  Freq.   Numeric  Label
                         1,807         0  No
                           749         1  Yes
                            64         .

-------------------------------------------------------------------------------------------------------------------------------------
bmicatn4                                                                                             WHO BMI categories (baseline)
-------------------------------------------------------------------------------------------------------------------------------------

                  type:  numeric (float)
                 label:  bmgrp2a

                 range:  [1,4]                        units:  1
         unique values:  4                        missing .:  45/2,620

            tabulation:  Freq.   Numeric  Label
                         1,633         1  18.5 - 24.99 kg/m.sq.
                           328         2  < 18.5 kg/m.sq.
                           404         3  25-29.99kg/m.sq.
                           210         4  >=30 kg/m.sq.
                            45         .
```

```
------------------------------------------------------------------------------------------------------------------------------------
parno                                                                                                                  Parish Number
------------------------------------------------------------------------------------------------------------------------------------

                  type:  numeric (float)
                 label:  parish

                 range:  [1,14]                       units:  1
         unique values:  14                       missing .:  1,369/2,620

              examples:  6     ST. ANN
                         14    ST. CATHERINE
                         .
                         .

------------------------------------------------------------------------------------------------------------------------------------
psu                                                                                                           Primary Sampling Unit
------------------------------------------------------------------------------------------------------------------------------------

                  type:  numeric (float)

                 range:  [1,88]                       units:  1
         unique values:  88                       missing .:  1,369/2,620

                  mean:   44.4269
              std. dev:   25.2771

           percentiles:        10%       25%       50%       75%       90%
                                 9        23        44        66        80

------------------------------------------------------------------------------------------------------------------------------------
pstratwt                                                                                                 Post-stratification Weights
------------------------------------------------------------------------------------------------------------------------------------

                  type:  numeric (float)

                 range:  [121.85185,294.61539]        units:  1.000e-06
         unique values:  28                       missing .:  1,369/2,620

                  mean:   189.216
              std. dev:   33.4214

           percentiles:        10%       25%       50%       75%       90%
                           156.451   167.571   184.204   201.786   219.941

------------------------------------------------------------------------------------------------------------------------------------
level2_psu                                                                                                          PSU - unique ID
------------------------------------------------------------------------------------------------------------------------------------

                  type:  numeric (float)
```

```
                  range:  [1,214]                      units:  1
          unique values:  213                      missing .:  84/2,620

                   mean:   68.4586
               std. dev:    60.076

            percentiles:        10%       25%       50%       75%       90%
                                  1         5        61       105       167

---------------------------------------------------------------------------------------------------------------------------------------
community                                                                                                                     COMMUNITY
---------------------------------------------------------------------------------------------------------------------------------------

                   type:  string (str31)

          unique values:  303                      missing "":  0/2,620

               examples:  "Cumberland"
                          "Greater Portmore"
                          "Lyssons"
                          "Rollington Town"

                warning:  variable has embedded blanks

---------------------------------------------------------------------------------------------------------------------------------------
parish                                                                                                                  Parish from MGI
---------------------------------------------------------------------------------------------------------------------------------------

                   type:  string (str13)

          unique values:  14                       missing "":  0/2,620

               examples:  "Manchester"
                          "St. Andrew"
                          "St. Ann"
                          "St. Catherine"

                warning:  variable has embedded blanks

---------------------------------------------------------------------------------------------------------------------------------------
population                                                                                                                   POPULATION
---------------------------------------------------------------------------------------------------------------------------------------

                   type:  numeric (long)

                  range:  [116,61267]                  units:  1
          unique values:  303                      missing .:  0/2,620

                   mean:   9351.38
               std. dev:   12195.2
```

```
         percentiles:        10%       25%       50%       75%       90%
                            1668      3016      5702     10552     17007

--------------------------------------------------------------------------------------------------------------------------------------
populationdensity                                                                                                 POPULATION DENSITY
--------------------------------------------------------------------------------------------------------------------------------------

                type:  numeric (int)

               range:  [10,9911]                    units:  1
       unique values:  280                      missing .:  0/2,620

                mean:   2380.51
            std. dev:      2785

         percentiles:        10%       25%       50%       75%       90%
                             149       270      1237      4075      7469

--------------------------------------------------------------------------------------------------------------------------------------
unemployment                                                                                                        UNEMPLOYMENT (%)
--------------------------------------------------------------------------------------------------------------------------------------

                type:  numeric (double)

               range:  [.44247788,44.830918]        units:  1.000e-08
       unique values:  306                      missing .:  0/2,620

                mean:   9.44282
            std. dev:   7.11433

         percentiles:        10%       25%       50%       75%       90%
                         4.66898   6.46644   8.05012    10.011   11.8899

--------------------------------------------------------------------------------------------------------------------------------------
incomeproxy                                                                                                             INCOME PROXY
--------------------------------------------------------------------------------------------------------------------------------------

                type:  numeric (byte)

               range:  [7,29]                       units:  1
       unique values:  23                       missing .:  0/2,620

                mean:   15.0061
            std. dev:   4.09061

         percentiles:        10%       25%       50%       75%       90%
                              10        12        15        17        20

--------------------------------------------------------------------------------------------------------------------------------------
poverty                                                                                                                POVERTY (%)
--------------------------------------------------------------------------------------------------------------------------------------
```

```
                  type:  numeric (double)

                 range:  [.034381,60.444245]          units:  1.000e-06
         unique values:  305                      missing .:  0/2,620

                  mean:   18.1931
              std. dev:   11.9084

           percentiles:        10%       25%       50%       75%       90%
                            4.09557   8.26031   17.2365   24.4439   37.4265

------------------------------------------------------------------------------------------------------------------------------------
urban                                                                                                                          URBAN
------------------------------------------------------------------------------------------------------------------------------------

                  type:  string (str3)

         unique values:  2                        missing "":  0/2,620

            tabulation:  Freq.  Value
                         1,330  "No"
                         1,290  "Yes"

------------------------------------------------------------------------------------------------------------------------------------
urban_n                                                                                                      urban community (MGI)
------------------------------------------------------------------------------------------------------------------------------------

                  type:  numeric (float)
                 label:  true

                 range:  [0,1]                        units:  1
         unique values:  2                        missing .:  0/2,620

            tabulation:  Freq.   Numeric  Label
                         1,330         0  No
                         1,290         1  Yes

------------------------------------------------------------------------------------------------------------------------------------
murders_2007                                                                                                            2007 MURDERS
------------------------------------------------------------------------------------------------------------------------------------

                  type:  numeric (byte)

                 range:  [0,33]                       units:  1
         unique values:  26                       missing .:  0/2,620

                  mean:   5.66298
              std. dev:   6.51293

           percentiles:        10%       25%       50%       75%       90%
```

```
                              0         1         4         9        15

-------------------------------------------------------------------------------------------------------------------------------------
murders_2008                                                                                                             2008 MURDERS
-------------------------------------------------------------------------------------------------------------------------------------

                  type:  numeric (byte)

                 range:  [0,34]                       units:  1
         unique values:  27                       missing .:  0/2,620

                  mean:   5.55763
              std. dev:   6.21789

           percentiles:        10%       25%       50%       75%       90%
                                 0         1         4         9        13

-------------------------------------------------------------------------------------------------------------------------------------
murders_2009                                                                                                             2009 MURDERS
-------------------------------------------------------------------------------------------------------------------------------------

                  type:  numeric (byte)

                 range:  [0,28]                       units:  1
         unique values:  24                       missing .:  0/2,620

                  mean:   4.99389
              std. dev:   5.47987

           percentiles:        10%       25%       50%       75%       90%
                                 0         0         3         8        12

-------------------------------------------------------------------------------------------------------------------------------------
nonmiss1                                                                                                                  (unlabeled)
-------------------------------------------------------------------------------------------------------------------------------------

                  type:  numeric (float)

                 range:  [2,5]                        units:  1
         unique values:  4                        missing .:  0/2,620

            tabulation:  Freq.  Value
                            35  2
                            28  3
                            12  4
                         2,545  5

-------------------------------------------------------------------------------------------------------------------------------------
_Isex_1                                                                                                                        sex==1
-------------------------------------------------------------------------------------------------------------------------------------
```

```
                  type:  numeric (byte)

                 range:  [0,1]                        units:  1
         unique values:  2                        missing .:  0/2,620

            tabulation:  Freq.  Value
                         1,485  0
                         1,135  1

-----------------------------------------------------------------------------------------------------------------------------------
include1                                                                                             Include in Neighbourhood models
-----------------------------------------------------------------------------------------------------------------------------------

                  type:  numeric (byte)
                 label:  true

                 range:  [0,1]                        units:  1
         unique values:  2                        missing .:  0/2,620

            tabulation:  Freq.   Numeric  Label
                            64         0  No
                         2,556         1  Yes

-----------------------------------------------------------------------------------------------------------------------------------
study                                                                                                    Study participant is from
-----------------------------------------------------------------------------------------------------------------------------------

                  type:  numeric (float)
                 label:  study

                 range:  [1,3]                        units:  1
         unique values:  3                        missing .:  0/2,620

            tabulation:  Freq.   Numeric  Label
                           894         1  1986 Birth Cohort
                         1,251         2  Youth Risk 2006
                           475         3  JHLS II

-----------------------------------------------------------------------------------------------------------------------------------
parish_mlm                                                                                                          PARISH for MLM
-----------------------------------------------------------------------------------------------------------------------------------

                  type:  numeric (long)
                 label:  parish

                 range:  [1,14]                       units:  1
         unique values:  14                       missing .:  0/2,620

              examples:  2     ST. ANDREW
                         2     ST. ANDREW
                         9     HANOVER
```

```
                         13    CLARENDON

-------------------------------------------------------------------------------------------------------------------------------------------
community_n                                                                                                            Community(numeric)
-------------------------------------------------------------------------------------------------------------------------------------------

                  type:  numeric (float)
                 label:  community_n

                 range:  [1,306]                      units:  1
         unique values:  306                      missing .:  0/2,620

              examples:  56    ST. ANDREW Greenwich Town/Newport West
                         113   ST. ANDREW Waterhouse
                         203   HANOVER Haddington
                         267   CLARENDON Trout Hall

-------------------------------------------------------------------------------------------------------------------------------------------
murder_total                                                                                                        Total Murders(2007-09)
-------------------------------------------------------------------------------------------------------------------------------------------

                  type:  numeric (float)

                 range:  [0,91]                       units:  1
         unique values:  50                       missing .:  0/2,620

                  mean:   16.2145
              std. dev:   16.5679

           percentiles:        10%       25%       50%       75%       90%
                                 0         3        11        25        36

-------------------------------------------------------------------------------------------------------------------------------------------
murder_rate                                                                                                    Murders per 1000 population
-------------------------------------------------------------------------------------------------------------------------------------------

                  type:  numeric (float)

                 range:  [0,29.230135]                units:  1.000e-08
         unique values:  257                      missing .:  0/2,620

                  mean:   2.22246
              std. dev:   2.91661

           percentiles:        10%       25%       50%       75%       90%
                                 0   .528785   1.47681   2.89775   4.65161

-------------------------------------------------------------------------------------------------------------------------------------------
comp1a                                                                                                          Scores for component 1
-------------------------------------------------------------------------------------------------------------------------------------------
```

```
                type:  numeric (float)

               range:  [-3.4373536,3.8301113]       units:  1.000e-10
       unique values:  306                      missing .:  0/2,620

                mean:   1.9e-09
            std. dev:   1.40622

         percentiles:        10%       25%       50%       75%       90%
                       -1.67984  -.855636  -.301289   .892217   1.56929

------------------------------------------------------------------------------------------------------------------------------------
comp2a                                                                                                       Scores for component 2
------------------------------------------------------------------------------------------------------------------------------------

                type:  numeric (float)

               range:  [-3.3983927,2.5662677]       units:  1.000e-10
       unique values:  306                      missing .:  0/2,620

                mean:  -3.3e-09
            std. dev:    1.1065

         percentiles:        10%       25%       50%       75%       90%
                       -1.25952  -.663639  -.143578   .618132   1.55851

------------------------------------------------------------------------------------------------------------------------------------
comp3a                                                                                                       Scores for component 3
------------------------------------------------------------------------------------------------------------------------------------

                type:  numeric (float)

               range:  [-1.5956604,8.7787609]       units:  1.000e-09
       unique values:  306                      missing .:  0/2,620

                mean:   1.3e-09
            std. dev:   1.00796

         percentiles:        10%       25%       50%       75%       90%
                       -.746463   -.51777  -.135609   .369415   .815071

------------------------------------------------------------------------------------------------------------------------------------
incomeproxy_rev                                                                                             Reversed INCOMEPROXY
------------------------------------------------------------------------------------------------------------------------------------

                type:  numeric (float)

               range:  [0,22]                       units:  1
       unique values:  23                       missing .:  0/2,620

                mean:   13.9939
```

```
           std. dev:   4.09061

        percentiles:        10%       25%       50%       75%       90%
                              9        12        14        17        19

-------------------------------------------------------------------------------------------------------------------------------------
incomeproxy_rev_std                                                                                        INCOMEPROXY_rev(Z score)
-------------------------------------------------------------------------------------------------------------------------------------

               type:  numeric (float)

              range:  [-3.4209819,1.9571928]       units:  1.000e-10
      unique values:  23                       missing .:  0/2,620

               mean:   5.2e-09
           std. dev:         1

        percentiles:        10%       25%       50%       75%       90%
                       -1.22082  -.487432   .001493    .73488   1.22381

-------------------------------------------------------------------------------------------------------------------------------------
poverty_std                                                                                                        POVERTY(Z score)
-------------------------------------------------------------------------------------------------------------------------------------

               type:  numeric (float)

              range:  [-1.5248615,3.5480108]       units:  1.000e-10
      unique values:  305                      missing .:  0/2,620

               mean:  -4.3e-09
           std. dev:         1

        percentiles:        10%       25%       50%       75%       90%
                       -1.18383  -.834095  -.080327   .524912   1.61511

-------------------------------------------------------------------------------------------------------------------------------------
unemployment_std                                                                                              UNEMPLOYMENT(Z score)
-------------------------------------------------------------------------------------------------------------------------------------

               type:  numeric (float)

              range:  [-1.2650996,4.974196]        units:  1.000e-11
      unique values:  306                      missing .:  0/2,620

               mean:   5.7e-09
           std. dev:         1

        percentiles:        10%       25%       50%       75%       90%
                       -.671017  -.418364   -.19576   .079864   .343965

-------------------------------------------------------------------------------------------------------------------------------------
```

```
populationdensity_std                                                              POPULATIONDENSITY(Z score)
-------------------------------------------------------------------------------------------------------------

                  type:  numeric (float)

                 range:  [-.85116774,2.7039435]       units:  1.000e-09
         unique values:  280                      missing .:  0/2,620

                  mean:   3.1e-09
              std. dev:         1

           percentiles:        10%       25%       50%       75%       90%
                          -.801258  -.757811  -.410594   .608435    1.8271

-------------------------------------------------------------------------------------------------------------
murder_rate_std                                                                        murder_rate(Z score)
-------------------------------------------------------------------------------------------------------------

                  type:  numeric (float)

                 range:  [-.76200044,9.2599678]       units:  1.000e-10
         unique values:  257                      missing .:  0/2,620

                  mean:   4.8e-09
              std. dev:         1

           percentiles:        10%       25%       50%       75%       90%
                             -.762  -.580699  -.255654   .231535   .832872

-------------------------------------------------------------------------------------------------------------
composite_sep                                                              Composite socioeconomic position
-------------------------------------------------------------------------------------------------------------

                  type:  numeric (float)

                 range:  [-6.5685654,9.7817793]       units:  1.000e-10
         unique values:  306                      missing .:  0/2,620

                  mean:   3.3e-08
              std. dev:   2.35439

           percentiles:        10%       25%       50%       75%       90%
                          -2.82564  -1.33002  -.320452   1.52129   3.47443

-------------------------------------------------------------------------------------------------------------
possession_cat_1986jbcs_old                                                    possession score categories
-------------------------------------------------------------------------------------------------------------

                  type:  numeric (float)
                 label:  possession_cat
```

```
          range:  [1,3]                        units:  1
  unique values:  3                        missing .:  1,728/2,620

    tabulation:  Freq.   Numeric  Label
                   136         1  15-17
                   494         2  10-14
                   262         3  0-9
                 1,728         .

--------------------------------------------------------------------------------------------------------------------------------------
possessn_cat3_jhls2                                                                       Possessions Categorised(excluding none)
--------------------------------------------------------------------------------------------------------------------------------------

           type:  numeric (byte)
          label:  thirds0

          range:  [0,2]                        units:  1
  unique values:  3                        missing .:  2,146/2,620

    tabulation:  Freq.   Numeric  Label
                   150         0  <= 8 items
                   161         1  9 - 11 items
                   163         2  12 - 18 items
                 2,146         .

--------------------------------------------------------------------------------------------------------------------------------------
posscat3b_jyrrbs                                                 Possessions Categorised (excluding owned by 90% or more)
--------------------------------------------------------------------------------------------------------------------------------------

           type:  numeric (float)
          label:  thirds2

          range:  [0,2]                        units:  1
  unique values:  3                        missing .:  1,370/2,620

    tabulation:  Freq.   Numeric  Label
                   442         0  <= 5 items
                   453         1  6-8 items
                   355         2  9-15 items
                 1,370         .

--------------------------------------------------------------------------------------------------------------------------------------
possession_score                                                                                    household possession score
--------------------------------------------------------------------------------------------------------------------------------------

           type:  numeric (float)

          range:  [0,17]                       units:  1
  unique values:  17                       missing .:  1,728/2,620

           mean:   11.2735
```

```
            std. dev:   3.08307

         percentiles:        10%       25%       50%       75%       90%
                               7         9        11        14        15

------------------------------------------------------------------------------------------------------------------------------------
posscat3b_1986jbcs                                                                                            Possessions Categorised
------------------------------------------------------------------------------------------------------------------------------------

                type:  numeric (byte)
               label:  thirds1a

               range:  [0,2]                        units:  1
       unique values:  3                        missing .:  1,728/2,620

          tabulation:  Freq.   Numeric  Label
                         350         0  <= 10 items
                         300         1  11-13 items
                         242         2  14-17 items
                       1,728         .

------------------------------------------------------------------------------------------------------------------------------------
possession_cat3                                                                                                Possession Categories
------------------------------------------------------------------------------------------------------------------------------------

                type:  numeric (float)
               label:  possession_cat3

               range:  [0,2]                        units:  1
       unique values:  3                        missing .:  4/2,620

          tabulation:  Freq.   Numeric  Label
                         942         0  Low
                         914         1  Middle
                         760         2  High
                           4         .

------------------------------------------------------------------------------------------------------------------------------------
averagetertiaryeducation                                                                                   AVERAGE TERTIARY EDUCATION
------------------------------------------------------------------------------------------------------------------------------------

                type:  numeric (double)

               range:  [.4225,56.67]                units:  .0001
       unique values:  304                      missing .:  0/2,620

                mean:   11.2342
            std. dev:   9.90215

         percentiles:        10%       25%       50%       75%       90%
                          2.5659     4.182    6.7911   16.4476   25.7244
```

```
------------------------------------------------------------------------------------------------------------------------------------
averagesecondaryeducation                                                                                AVERAGE SECONDARY EDUCATION
------------------------------------------------------------------------------------------------------------------------------------

                  type:  numeric (double)

                 range:  [23.235,68.84]               units:  .0001
         unique values:  305                      missing .:  0/2,620

                  mean:   48.1106
              std. dev:   6.46505

           percentiles:        10%       25%       50%       75%       90%
                             38.78   45.7433     48.35   52.3759   56.4491

------------------------------------------------------------------------------------------------------------------------------------
averageprimaryeducation                                                                                    AVERAGE PRIMARY EDUCATION
------------------------------------------------------------------------------------------------------------------------------------

                  type:  numeric (double)

                 range:  [4.3138,62.595]              units:  .0001
         unique values:  306                      missing .:  0/2,620

                  mean:   30.4456
              std. dev:   9.44641

           percentiles:        10%       25%       50%       75%       90%
                           19.2777   24.6747   28.9719   36.0342   44.2898

------------------------------------------------------------------------------------------------------------------------------------
of2bedroomdwellings                                                                                        % OF 2 BEDROOM DWELLINGS
------------------------------------------------------------------------------------------------------------------------------------

                  type:  numeric (double)

                 range:  [.83,26.34]                  units:  .0001
         unique values:  305                      missing .:  0/2,620

                  mean:   8.38315
              std. dev:   3.62801

           percentiles:        10%       25%       50%       75%       90%
                           4.80665    6.3356    7.4677   10.1033   12.9705

------------------------------------------------------------------------------------------------------------------------------------
dependencyratio                                                                                                     DEPENDENCY RATIO
------------------------------------------------------------------------------------------------------------------------------------

                  type:  numeric (double)
```

```
          range:  [.304,1.159]                 units:  .001
  unique values:  223                      missing .:  0/2,620

           mean:   .718715
       std. dev:   .123756

    percentiles:        10%       25%       50%       75%       90%
                       .554      .628      .715      .806      .876

-------------------------------------------------------------------------------------------------------------------------------------
averagetertiaryeducation_rev                                                                   Per cent without teriary education
-------------------------------------------------------------------------------------------------------------------------------------

           type:  numeric (float)

          range:  [43.33,99.5775]              units:  .0001
  unique values:  304                      missing .:  0/2,620

           mean:   88.7658
       std. dev:   9.90215

    percentiles:        10%       25%       50%       75%       90%
                    74.2756   83.5524   93.2089    95.818   97.4341

-------------------------------------------------------------------------------------------------------------------------------------
of2bedroomdwellings_rev                                                                                              House size proxy
-------------------------------------------------------------------------------------------------------------------------------------

           type:  numeric (float)

          range:  [.03796507,1.2048193]        units:  1.000e-09
  unique values:  305                      missing .:  0/2,620

           mean:   .155457
       std. dev:   .144885

    percentiles:        10%       25%       50%       75%       90%
                    .077098   .098978    .13391   .157838   .208048

-------------------------------------------------------------------------------------------------------------------------------------
of2bedroomdwellings_rev2                                                                                                  (unlabeled)
-------------------------------------------------------------------------------------------------------------------------------------

           type:  numeric (float)

          range:  [-26.34,-.83]                units:  .0001
  unique values:  305                      missing .:  0/2,620

           mean:  -8.38315
       std. dev:   3.62801
```

```
     percentiles:        10%       25%       50%       75%       90%
                    -12.9705  -10.1033   -7.4677   -6.3356  -4.80665

------------------------------------------------------------------------------------------------------------------------------------
of2bedroomdwellings_rev3                                                                                      of2bedroomdwellings reversed
------------------------------------------------------------------------------------------------------------------------------------

            type:  numeric (float)

           range:  [.83,26.34]                  units:  .0001
   unique values:  305                      missing .:  0/2,620

            mean:   18.7868
       std. dev:   3.62801

     percentiles:        10%       25%       50%       75%       90%
                     14.1995   17.0667   19.7023   20.8344   22.3633

------------------------------------------------------------------------------------------------------------------------------------
pca_comp1                                                                                               PCA (higher) SES (component 1)
------------------------------------------------------------------------------------------------------------------------------------

            type:  numeric (float)

           range:  [-4.6062326,5.3988872]       units:  1.000e-09
   unique values:  307                      missing .:  0/2,620

            mean:  -8.8e-10
       std. dev:   1.80725

     percentiles:        10%       25%       50%       75%       90%
                    -2.18177  -1.16803  -.400786   1.30642   3.03212

------------------------------------------------------------------------------------------------------------------------------------
pca_comp2                                                                                                PCA (lower) SES (component 2)
------------------------------------------------------------------------------------------------------------------------------------

            type:  numeric (float)

           range:  [-3.9673362,4.3185782]       units:  1.000e-11
   unique values:  307                      missing .:  0/2,620

            mean:   1.8e-09
       std. dev:   1.19533

     percentiles:        10%       25%       50%       75%       90%
                    -1.01495  -.572826  -.171759   .430491   1.13523

------------------------------------------------------------------------------------------------------------------------------------
pca_comp1_tert                                                                                                   3 quantiles of pca_comp1
```

```
------------------------------------------------------------------------------------------------------------------------------------

                  type:  numeric (byte)

                 range:  [1,3]                        units:  1
         unique values:  3                        missing .:  0/2,620

            tabulation:  Freq.  Value
                           884  1
                           872  2
                           864  3

------------------------------------------------------------------------------------------------------------------------------------
pca_comp2_tert                                                                                               3 quantiles of pca_comp2
------------------------------------------------------------------------------------------------------------------------------------

                  type:  numeric (byte)

                 range:  [1,3]                        units:  1
         unique values:  3                        missing .:  0/2,620

            tabulation:  Freq.  Value
                           882  1
                           886  2
                           852  3

------------------------------------------------------------------------------------------------------------------------------------
miss_age                                                                                                                  missing age
------------------------------------------------------------------------------------------------------------------------------------

                  type:  numeric (float)
                 label:  true

                 range:  [0,1]                        units:  1
         unique values:  2                        missing .:  0/2,620

            tabulation:  Freq.   Numeric  Label
                         2,619         0  No
                             1         1  Yes

------------------------------------------------------------------------------------------------------------------------------------
miss_weight                                                                                                            missing weight
------------------------------------------------------------------------------------------------------------------------------------

                  type:  numeric (float)
                 label:  true

                 range:  [0,1]                        units:  1
         unique values:  2                        missing .:  0/2,620

            tabulation:  Freq.   Numeric  Label
```

```
                    2,582         0  No
                       38         1  Yes

-------------------------------------------------------------------------------------------------------------------------------------
miss_height_cm                                                                                                     missing height_cm
-------------------------------------------------------------------------------------------------------------------------------------

                  type:  numeric (float)
                 label:  true

                 range:  [0,1]                        units:  1
         unique values:  2                        missing .:  0/2,620

            tabulation:  Freq.   Numeric  Label
                         2,577         0  No
                            43         1  Yes

-------------------------------------------------------------------------------------------------------------------------------------
miss_bmi                                                                                                                 missing bmi
-------------------------------------------------------------------------------------------------------------------------------------

                  type:  numeric (float)
                 label:  true

                 range:  [0,1]                        units:  1
         unique values:  2                        missing .:  0/2,620

            tabulation:  Freq.   Numeric  Label
                         2,575         0  No
                            45         1  Yes

-------------------------------------------------------------------------------------------------------------------------------------
miss_waistcirc                                                                                           missing waist circumference
-------------------------------------------------------------------------------------------------------------------------------------

                  type:  numeric (float)
                 label:  true

                 range:  [0,1]                        units:  1
         unique values:  2                        missing .:  0/2,620

            tabulation:  Freq.   Numeric  Label
                         2,576         0  No
                            44         1  Yes

-------------------------------------------------------------------------------------------------------------------------------------
miss_fastglu                                                                                                 missing fasting glucose
-------------------------------------------------------------------------------------------------------------------------------------

                  type:  numeric (float)
                 label:  true
```

```
                range:  [0,1]                        units:  1
        unique values:  2                        missing .:  0/2,620

           tabulation:  Freq.   Numeric  Label
                        2,430         0  No
                          190         1  Yes

-------------------------------------------------------------------------------------------------------------------------------------------
miss_fast_chol                                                                                             missing fasting cholesterol
-------------------------------------------------------------------------------------------------------------------------------------------

                 type:  numeric (float)
                label:  true

                range:  [0,1]                        units:  1
        unique values:  2                        missing .:  0/2,620

           tabulation:  Freq.   Numeric  Label
                        2,422         0  No
                          198         1  Yes

-------------------------------------------------------------------------------------------------------------------------------------------
miss_possession_cat3                                                                                              miss_possession_cat3
-------------------------------------------------------------------------------------------------------------------------------------------

                 type:  numeric (float)
                label:  true

                range:  [0,1]                        units:  1
        unique values:  2                        missing .:  0/2,620

           tabulation:  Freq.   Numeric  Label
                        2,616         0  No
                            4         1  Yes

-------------------------------------------------------------------------------------------------------------------------------------------
miss_imputed                                                                                       missing values for imputed variables
-------------------------------------------------------------------------------------------------------------------------------------------

                 type:  numeric (float)
                label:  true

                range:  [0,1]                        units:  1
        unique values:  2                        missing .:  0/2,620

           tabulation:  Freq.   Numeric  Label
                        2,373         0  No
                          247         1  Yes

-------------------------------------------------------------------------------------------------------------------------------------------
```

```
std_poverty                                                                        Standardized values of (poverty)
-------------------------------------------------------------------------------------------------------------------

                  type:  numeric (float)

                 range:  [-1.5248615,3.5480108]       units:  1.000e-10
         unique values:  305                      missing .:  0/2,620

                  mean:  -4.3e-09
             std. dev:         1

           percentiles:        10%       25%       50%       75%       90%
                          -1.18383  -.834095  -.080327   .524912   1.61511

-------------------------------------------------------------------------------------------------------------------
std_unemployment                                                              Standardized values of (unemployment)
-------------------------------------------------------------------------------------------------------------------

                  type:  numeric (float)

                 range:  [-1.2650996,4.974196]        units:  1.000e-11
         unique values:  306                      missing .:  0/2,620

                  mean:   5.7e-09
             std. dev:         1

           percentiles:        10%       25%       50%       75%       90%
                          -.671017  -.418364   -.19576   .079864   .343965

-------------------------------------------------------------------------------------------------------------------
std_dependencyratio                                                        Standardized values of (dependencyratio)
-------------------------------------------------------------------------------------------------------------------

                  type:  numeric (float)

                 range:  [-3.3510766,3.5577016]       units:  1.000e-10
         unique values:  223                      missing .:  0/2,620

                  mean:   1.4e-09
             std. dev:         1

           percentiles:        10%       25%       50%       75%       90%
                          -1.33097  -.733013  -.030015   .705305   1.27094

-------------------------------------------------------------------------------------------------------------------
std_populationdensity                                                    Standardized values of (populationdensity)
-------------------------------------------------------------------------------------------------------------------

                  type:  numeric (float)

                 range:  [-.85116774,2.7039435]       units:  1.000e-09
```

```
        unique values:  280                      missing .:  0/2,620

                 mean:   3.1e-09
             std. dev:         1

          percentiles:        10%       25%       50%       75%       90%
                         -.801258  -.757811  -.410594   .608435    1.8271

------------------------------------------------------------------------------------------------------------------------------------------
std_of2bedroomdwellings                                                               Standardized values of (of2bedroomdwellings)
------------------------------------------------------------------------------------------------------------------------------------------

                 type:  numeric (float)

                range:  [-2.0818985,4.9495001]       units:  1.000e-10
        unique values:  305                      missing .:  0/2,620

                 mean:   3.7e-09
             std. dev:         1

          percentiles:        10%       25%       50%       75%       90%
                         -.985802  -.564373  -.252329   .474129   1.26442

------------------------------------------------------------------------------------------------------------------------------------------
std_averagetertiaryeducation                                                     Standardized values of (averagetertiaryeducation)
------------------------------------------------------------------------------------------------------------------------------------------

                 type:  numeric (float)

                range:  [-1.0918517,4.5884776]       units:  1.000e-09
        unique values:  304                      missing .:  0/2,620

                 mean:  -3.2e-09
             std. dev:         1

          percentiles:        10%       25%       50%       75%       90%
                         -.875394  -.712187  -.448699   .526493   1.46334

------------------------------------------------------------------------------------------------------------------------------------------
pca_comp1_rot                                                                                              Scores for component 1
------------------------------------------------------------------------------------------------------------------------------------------

                 type:  numeric (float)

                range:  [-4.2353492,6.1199603]       units:  1.000e-09
        unique values:  307                      missing .:  0/2,620

                 mean:   3.7e-09
             std. dev:   1.77104

          percentiles:        10%       25%       50%       75%       90%
```

```
                    -2.04193  -1.17982  -.454051   1.17931   2.53513

--------------------------------------------------------------------------------------------------------------------------------------
pca_comp2_rot                                                                                                  Scores for component 2
--------------------------------------------------------------------------------------------------------------------------------------

                  type:  numeric (float)

                 range:  [-2.4292235,4.8002477]       units:  1.000e-09
         unique values:  307                        missing .:  0/2,620

                  mean:   6.2e-09
             std. dev:   1.24837

           percentiles:        10%       25%       50%       75%       90%
                           -1.10876  -.714972  -.262889   .342759   1.04993

--------------------------------------------------------------------------------------------------------------------------------------
mn23sbp_tert                                                                                                 Tertiles for mn23sbp
--------------------------------------------------------------------------------------------------------------------------------------

                  type:  numeric (byte)
                 label:  tert

                 range:  [1,3]                        units:  1
         unique values:  3                          missing .:  63/2,620

           tabulation:  Freq.   Numeric  Label
                          927         1  Tertile 1
                          826         2  Tertile 2
                          804         3  Tertile 3
                           63         .

--------------------------------------------------------------------------------------------------------------------------------------
mn23dbp_tert                                                                                                 Tertiles for mn23dbp
--------------------------------------------------------------------------------------------------------------------------------------

                  type:  numeric (byte)
                 label:  tert

                 range:  [1,3]                        units:  1
         unique values:  3                          missing .:  64/2,620

           tabulation:  Freq.   Numeric  Label
                          857         1  Tertile 1
                          902         2  Tertile 2
                          797         3  Tertile 3
                           64         .

--------------------------------------------------------------------------------------------------------------------------------------
d10                                                                  Juici Patties,Tastee, Pizza Hut, Kentucky Fried Chicken?
```

```
--------------------------------------------------------------------------------------------------------------------------------------

                type:  numeric (byte)

               range:  [1,99]                       units:  1
       unique values:  8                        missing .:  1,789/2,620

          tabulation:  Freq.  Value
                          88  1
                         379  2
                         165  3
                          86  4
                          25  5
                          81  6
                           6  88
                           1  99
                       1,789  .

--------------------------------------------------------------------------------------------------------------------------------------
phys_cat                                                                                                  Physical Activity Categories
--------------------------------------------------------------------------------------------------------------------------------------

                type:  numeric (float)
               label:  physlev

               range:  [1,3]                        units:  1
       unique values:  3                        missing .:  1,369/2,620

          tabulation:  Freq.   Numeric  Label
                         389         1  Low
                         278         2  Moderate
                         584         3  High
                       1,369         .

--------------------------------------------------------------------------------------------------------------------------------------
fastfdcat1                                                                                                  Fast-food Consumption (2)
--------------------------------------------------------------------------------------------------------------------------------------

                type:  numeric (float)
               label:  jkfdcat

               range:  [0,2]                        units:  1
       unique values:  3                        missing .:  1,381/2,620

          tabulation:  Freq.   Numeric  Label
                       1,047         0  Seldom
                         111         1  Frequent
                          81         2  Excessive
                       1,381         .

--------------------------------------------------------------------------------------------------------------------------------------
```

```
source                                                                    (unlabeled)
-------------------------------------------------------------------------------------

                  type:  numeric (byte)
                 label:  source

                 range:  [0,2]                        units:  1
         unique values:  3                        missing .:  0/2,620

            tabulation:  Freq.   Numeric  Label
                         1,251         0  JYRRBS
                           894         1  1986 BC
                           475         2  JHLSII

-------------------------------------------------------------------------------------
q62nut                                                                  eat fast food
-------------------------------------------------------------------------------------

                  type:  numeric (byte)
                 label:  q62nut

                 range:  [0,99]                       units:  1
         unique values:  7                        missing .:  1,736/2,620

            tabulation:  Freq.   Numeric  Label
                           263         0  < once/week
                           425         1  1-2 times/week
                           122         2  3-4 times/week
                            59         3  5-6 times/week
                            13         4  everyday
                             1         9  dk
                             1        99  nr
                         1,736         .

-------------------------------------------------------------------------------------
phys_act_time                                                  Physical Activity Time
-------------------------------------------------------------------------------------

                  type:  numeric (float)

                 range:  [0,40]                       units:  .1
         unique values:  26                       missing .:  1,728/2,620

                  mean:   3.31054
             std. dev:    5.34935

           percentiles:        10%       25%       50%       75%       90%
                                 0         0       1.5       3.5         9

-------------------------------------------------------------------------------------
phys_act_level                                                Physical Activity Level
```

```
-----------------------------------------------------------------------------------------------------------------------------------

                  type:  numeric (float)
                 label:  phys_act_level

                 range:  [0,2]                        units:  1
         unique values:  3                        missing .:  1,728/2,620

            tabulation:  Freq.   Numeric  Label
                           304         0  Low PAL
                           373         1  Moderate PAL
                           215         2  High PAL
                         1,728         .

-----------------------------------------------------------------------------------------------------------------------------------
pa_local                                                                                                          (Local) PA Levels
-----------------------------------------------------------------------------------------------------------------------------------

                  type:  numeric (byte)
                 label:  active

                 range:  [1,4]                        units:  1
         unique values:  4                        missing .:  2,147/2,620

            tabulation:  Freq.   Numeric  Label
                           127         1  High activity
                           108         2  Medium activity
                            75         3  Low activity
                           163         4  Inactive
                         2,147         .

-----------------------------------------------------------------------------------------------------------------------------------
fastfdfr1                                                                                                       Fast Food Frequency
-----------------------------------------------------------------------------------------------------------------------------------

                  type:  numeric (float)
                 label:  eatfastfd1

                 range:  [0,6]                        units:  1
         unique values:  7                        missing .:  2,146/2,620

            tabulation:  Freq.   Numeric  Label
                           203         0  Don't eat out
                            43         1  <1x/wk
                           118         2  1x/wk
                            51         3  2x/wk
                            25         4  3x/wk
                            12         5  4x/wk
                            22         6  >=5x/wk
                         2,146         .
```

```
--------------------------------------------------------------------------------------------------------------------------------------
short_ipaq_cat                                                                                            Physical activity categories
--------------------------------------------------------------------------------------------------------------------------------------

                  type:  numeric (float)
                 label:  short_ipaq_cat

                 range:  [1,3]                        units:  1
         unique values:  3                        missing .:  2,212/2,620

            tabulation:  Freq.   Numeric  Label
                           282         1  Low
                           112         2  Moderate
                            14         3  High
                         2,212         .

--------------------------------------------------------------------------------------------------------------------------------------
phys_act2020                                                                                                  Physical Activity Levels
--------------------------------------------------------------------------------------------------------------------------------------

                  type:  numeric (float)
                 label:  phys_act2020

                 range:  [0,2]                        units:  1
         unique values:  3                        missing .:  4/2,620

            tabulation:  Freq.   Numeric  Label
                           926         0  High
                           759         1  Moderate
                           931         2  Low
                             4         .

--------------------------------------------------------------------------------------------------------------------------------------
fastfood_cat2020                                                                                       Fast Food Consumption frequency
--------------------------------------------------------------------------------------------------------------------------------------

                  type:  numeric (float)
                 label:  fastfood_cat2020

                 range:  [0,2]                        units:  1
         unique values:  3                        missing .:  25/2,620

            tabulation:  Freq.   Numeric  Label
                         2,099         0  <= 2 times/wk
                           309         1  3-4 times/wk
                           187         2  >= 5 times/wk
                            25         .

--------------------------------------------------------------------------------------------------------------------------------------
bmi_std                                                                                                BMI Z scores (study-specific)
--------------------------------------------------------------------------------------------------------------------------------------
```

```
                  type:  numeric (float)

                 range:  [-1.8085015,7.0631218]       units:  1.000e-11
         unique values:  2,552                    missing .:  45/2,620

                  mean:  -8.9e-10
              std. dev:   .999611

           percentiles:        10%       25%       50%       75%       90%
                          -.930043  -.640861  -.235803   .388643   1.19152

-------------------------------------------------------------------------------------------------------------------------------
bmi_std1                                                                                                    BMI Z scores (study 1)
-------------------------------------------------------------------------------------------------------------------------------

                  type:  numeric (float)

                 range:  [-1.8085015,5.4614501]       units:  1.000e-11
         unique values:  884                      missing .:  1,729/2,620

                  mean:  -7.1e-10
              std. dev:        1

           percentiles:        10%       25%       50%       75%       90%
                          -.908924  -.641437  -.258537   .388643   1.19498

-------------------------------------------------------------------------------------------------------------------------------
bmi_std2                                                                                                    BMI Z scores (study 2)
-------------------------------------------------------------------------------------------------------------------------------

                  type:  numeric (float)

                 range:  [-1.6779333,7.0631218]       units:  1.000e-11
         unique values:  1,206                    missing .:  1,398/2,620

                  mean:  -1.1e-09
              std. dev:        1

           percentiles:        10%       25%       50%       75%       90%
                          -.937817  -.635344  -.220904   .372657   1.16812

-------------------------------------------------------------------------------------------------------------------------------
bmi_std3                                                                                                    BMI Z scores (study 3)
-------------------------------------------------------------------------------------------------------------------------------

                  type:  numeric (float)

                 range:  [-1.4008383,6.3245783]       units:  1.000e-10
         unique values:  462                      missing .:  2,158/2,620
```

```
                  mean:  -6.7e-10
              std. dev:         1

           percentiles:        10%       25%       50%       75%       90%
                          -.970899  -.646647  -.250323   .448986   1.24284

-------------------------------------------------------------------------------------------------------------------------------------
pca_comp1_std                                                                                                 pca_comp1 standardised
-------------------------------------------------------------------------------------------------------------------------------------

                  type:  numeric (float)

                 range:  [-2.548749,2.9873455]        units:  1.000e-09
         unique values:  307                      missing .:  0/2,620

                  mean:   1.6e-09
              std. dev:         1

           percentiles:        10%       25%       50%       75%       90%
                          -1.20723  -.646299  -.221765   .722877   1.67775

-------------------------------------------------------------------------------------------------------------------------------------
pca_comp2_std                                                                                                 pca_comp2 standardised
-------------------------------------------------------------------------------------------------------------------------------------

                  type:  numeric (float)

                 range:  [-3.3190186,3.6128628]       units:  1.000e-11
         unique values:  307                      missing .:  0/2,620

                  mean:  -3.1e-10
              std. dev:         1

           percentiles:        10%       25%       50%       75%       90%
                          -.849091  -.479218  -.143691   .360143   .949716

-------------------------------------------------------------------------------------------------------------------------------------
pca_comp1_std_sqd                                                                                            pca_comp1_std squared
-------------------------------------------------------------------------------------------------------------------------------------

                  type:  numeric (float)

                 range:  [.00006666,8.9242325]        units:  1.000e-12
         unique values:  307                      missing .:  0/2,620

                  mean:   .999618
              std. dev:    1.1966

           percentiles:        10%       25%       50%       75%       90%
                           .032528   .155538   .467285   1.54565   2.85415
```

```
-------------------------------------------------------------------------------------------------------------------------------------
pca_comp2_std_sqd                                                                                             pca_comp2_std squared
-------------------------------------------------------------------------------------------------------------------------------------

                  type:  numeric (float)

                 range:  [1.498e-09,13.052778]        units:  1.000e-16
         unique values:  307                      missing .:  0/2,620

                  mean:   .999618
              std. dev:   2.51201

           percentiles:        10%       25%       50%       75%       90%
                            .011454   .040746   .211045   .570219   1.88568

-------------------------------------------------------------------------------------------------------------------------------------
pca_comp1_std_cubed                                                                                             pca_comp1_std cubed
-------------------------------------------------------------------------------------------------------------------------------------

                  type:  numeric (float)

                 range:  [-16.556982,26.659767]       units:  1.000e-14
         unique values:  307                      missing .:  0/2,620

                  mean:    .44223
              std. dev:   2.92197

           percentiles:        10%       25%       50%       75%       90%
                           -1.75941  -.269961  -.010906    .37774    4.7233

-------------------------------------------------------------------------------------------------------------------------------------
pca_comp2_std_cubed                                                                                             pca_comp2_std cubed
-------------------------------------------------------------------------------------------------------------------------------------

                  type:  numeric (float)

                 range:  [-36.561924,47.157894]       units:  1.000e-20
         unique values:  307                      missing .:  0/2,620

                  mean:   1.45447
              std. dev:   8.77142

           percentiles:        10%       25%       50%       75%       90%
                           -.612158  -.110053  -.002969   .046711   .856607

-------------------------------------------------------------------------------------------------------------------------------------
fitted_sbp                                                                                                            Fitted values
-------------------------------------------------------------------------------------------------------------------------------------

                  type:  numeric (float)
```

```
           range:  [104.12885,112.68096]        units:  1.000e-06
   unique values:  307                      missing .:  63/2,620

            mean:   111.494
        std. dev:   .946331

     percentiles:        10%       25%       50%       75%       90%
                     110.665   111.228   111.624   112.066   112.447

-------------------------------------------------------------------------------------------------------------------------------------
pca_comp2_stdm1                                                                                           pca_comp2_std: (.,-1)
-------------------------------------------------------------------------------------------------------------------------------------

            type:  numeric (float)

           range:  [-3.3190186,-1]              units:  1.000e-07
   unique values:  31                       missing .:  0/2,620

            mean:  -1.04779
        std. dev:   .209509

     percentiles:        10%       25%       50%       75%       90%
                          -1        -1        -1        -1        -1

-------------------------------------------------------------------------------------------------------------------------------------
pca_comp2_std0                                                                                            pca_comp2_std: (-1,0)
-------------------------------------------------------------------------------------------------------------------------------------

            type:  numeric (float)

           range:  [0,1]                        units:  1.000e-09
   unique values:  173                      missing .:  0/2,620

            mean:   .715524
        std. dev:   .333323

     percentiles:        10%       25%       50%       75%       90%
                     .150909   .520782   .856309         1         1

-------------------------------------------------------------------------------------------------------------------------------------
pca_comp2_std1                                                                                             pca_comp2_std: (0,1)
-------------------------------------------------------------------------------------------------------------------------------------

            type:  numeric (float)

           range:  [0,1]                        units:  1.000e-10
   unique values:  90                       missing .:  0/2,620

            mean:   .213849
        std. dev:   .338333
```

```
      percentiles:        10%       25%       50%       75%       90%
                            0         0         0   .360143   .949716

-----------------------------------------------------------------------------------------------------------------------------------
pca_comp2_std4                                                                                                pca_comp2_std: (1,.)
-----------------------------------------------------------------------------------------------------------------------------------

             type:  numeric (float)

            range:  [0,2.6128628]                units:  1.000e-09
    unique values:  19                       missing .:  0/2,620

             mean:   .118415
         std. dev:   .504958

      percentiles:        10%       25%       50%       75%       90%
                            0         0         0         0         0

-----------------------------------------------------------------------------------------------------------------------------------
num_missing                                                                  number of missing values for included variables
-----------------------------------------------------------------------------------------------------------------------------------

             type:  numeric (float)

            range:  [0,6]                        units:  1
    unique values:  7                        missing .:  64/2,620

       tabulation:  Freq.  Value
                    2,325  0
                      118  1
                      101  2
                        8  3
                        1  4
                        2  5
                        1  6
                       64  .
```
